# Intelligence outcome of pediatric intensive care unit survivors: a systematic meta-analysis and meta-regression

**DOI:** 10.1186/s12916-022-02390-5

**Published:** 2022-06-01

**Authors:** Eleonore S. V. de Sonnaville, Marsh Kӧnigs, Ouke van Leijden, Hennie Knoester, Job B. M. van Woensel, Jaap Oosterlaan

**Affiliations:** 1 Amsterdam UMC, University of Amsterdam, Emma Children’s Hospital, Department of Pediatric Intensive Care, Amsterdam Reproduction and Development research institute, Meibergdreef 9, Amsterdam, The Netherlands; 2 Amsterdam UMC, University of Amsterdam, Emma Children’s Hospital, Department of Pediatrics, Amsterdam UMC Follow-Me Program & Emma Neuroscience Group, Amsterdam Reproduction and Development research institute, Meibergdreef 9, Amsterdam, The Netherlands

**Keywords:** Children, Pediatric intensive care, Outcome, Intelligence, Cognitive, IQ, Development, Meta-analysis, Meta-regression

## Abstract

**Background:**

Long-term morbidity after pediatric intensive care unit (PICU) admission is a growing concern. Both critical illness and accompanying PICU treatments may impact neurocognitive development as assessed by its gold standard measure; intelligence. This meta-analysis and meta-regression quantifies intelligence outcome after PICU admission and explores risk factors for poor intelligence outcome.

**Methods:**

PubMed, Embase, CINAHL and PsycINFO were searched for relevant studies, published from database inception until September 7, 2021. Using random-effects meta-analysis, we calculated the standardized mean difference in full-scale intelligence quotient (FSIQ) between PICU survivors and controls across all included studies and additionally distinguishing between PICU subgroups based on indications for admission. Relation between demographic and clinical risk factors and study’s FSIQ effect sizes was investigated using random-effects meta-regression analysis.

**Results:**

A total of 123 articles was included, published between 1973 and 2021, including 8,119 PICU survivors and 1,757 controls. We found 0.47 SD (7.1 IQ-points) lower FSIQ scores in PICU survivors compared to controls (95%CI -0.55 to -0.40, *p* < .001). All studied PICU subgroups had lower FSIQ compared to controls (range 0.38–0.88 SD). Later year of PICU admission (range 1972–2016) and longer PICU stay were related to greater FSIQ impairment (R^*2*^ = 21%, 95%CI -0.021 to -0.007, *p* < .001 and R^*2*^ = 2%, 95%CI -0.027 to -0.002, *p* = .03, respectively), whereas male sex and higher rate of survivors were related to smaller FSIQ impairment (R^*2*^ = 5%, 95%CI 0.001 to 0.014, *p* = .03 and R^*2*^ = 11%, 95%CI 0.006 to 0.022, *p* < .001, respectively). Meta-regression in PICU subgroups showed that later year of PICU admission was related to greater FSIQ impairment in children admitted after cardiac surgery and heart- or heart–lung transplantation. Male sex was related to smaller FSIQ impairment in children admitted after cardiac surgery. Older age at PICU admission and older age at follow-up were related to smaller FSIQ impairment in children admitted after heart- or heart–lung transplantation.

**Conclusions:**

PICU survivors, distinguished in a wide range of subgroups, are at risk of intelligence impairment. Length of PICU stay, female sex and lower rate of survivors were related to greater intelligence impairment. Intelligence outcome has worsened over the years, potentially reflecting the increasing percentage of children surviving PICU admission.

**Supplementary Information:**

The online version contains supplementary material available at 10.1186/s12916-022-02390-5.

## Background

Due to major advances in pediatric critical care, the survival rate of children admitted to the pediatric intensive care unit (PICU) has increased dramatically in the past decades [1, 2]. Nevertheless, long-term morbidity after PICU admission is a growing concern [2–8]. Both the critical illness and the accompanying PICU treatments may impact neurocognitive development as assessed by its gold standard measure intelligence. Intelligence is associated with important life outcomes, such as physical and mental health [9, 10], academic achievement [11], socioeconomic success [12], and life chances in general [10]. These findings highlight intelligence as an important outcome after PICU admission.

Several pathophysiological mechanisms are proposed that may impair long-term intelligence outcome of critically ill patients, including hypoxia, metabolic derangements such as glucose dysregulation, ischemia, inflammation, hypotension and delirium [13–15]. These mechanisms may be influenced by the underlying disease [16], critical illness [17] and associated treatments at the PICU [18]. A previous systematic review [19], including 12 studies of which the majority reported on children admitted for sepsis, identified an increased risk of intelligence impairment among PICU survivors. However, meta-analytic quantification of the magnitude of intelligence impairment was not performed, and the available data did not allow to systematically explore predictive factors of intelligence outcome. Given the distinct heterogeneity in the PICU population (e.g. admission indication, associated treatments and age), it is of great importance to determine intelligence outcome of PICU survivors and identify risk factors for poor intelligence outcome.

The current meta-analysis and meta-regression aims to [1] quantify intelligence outcome of PICU survivors; and [2] explore risk factors for poor intelligence outcome. The results of this study will provide valuable information for prognosis and early identification of children at risk for neurocognitive impairment, facilitating determination of the need for follow-up and/or early intervention after PICU discharge.

## Methods

### Study selection

Inclusion criteria for studies were: [1] the study sample consisted of PICU survivors who had been admitted to a general PICU or specialized PICU; [2] full-scale intelligence quotient (FSIQ) was assessed after PICU hospitalization using (short-forms of) standardized and validated tests; and [3] published in a peer-reviewed journal. Exclusion criteria were: [1] the study reported insufficient data to allow calculation of the individual study’s effect size; [2] the study sample comprised > 5% patients suffering from hereditary syndromes with known intelligence impairment (e.g. Down syndrome); [3] part of the sample comprised children hospitalized at other facilities than the PICU; [4] sample size < 10 children; [5] the study was written in Chinese; [6] the study could not be retrieved via our research institutes or via the authors. In case multiple articles reported on (partly) overlapping cohorts, only one article was selected that reported on (in order of importance): [1] the longest follow-up period; [2] the largest sample size; [3] the most extensive set of risk factors for intelligence impairment.

PubMed, Embase, CINAHL and PsycINFO were searched, without language or date restriction (last search September 7, 2021), using combinations of search terms relating to the [1] PICU, [2] children and [3] intelligence. The complete search strategy is provided in Additional file 1. Studies identified by our search were reviewed by two independent authors and disagreements were solved through discussion or by consulting a third author. Reference lists of the included studies were screened. This meta-analysis was conducted according to PRISMA guidelines.[20] The review protocol was registered in the International Prospective Register of Systematic Reviews, PROSPERO (#CRD42020197282) [21].

### Outcomes and covariates

We extracted descriptives on FSIQ of the PICU sample (and healthy control sample, if available) and extracted a broad range of demographic and clinical variables as potential risk factors for poor intelligence outcome. The extracted variables were variables reported at least once in the ten most recently published included articles (see Additional file 1: Table S1). In addition, in articles focusing on cardiac surgery and heart- or heart–lung transplantation, we also extracted the percentage of patients receiving cardiopulmonary bypass (CPB) and/or deep-hypothermic circulatory arrest (DHCA) during surgery, CPB duration during surgery, and the percentage of patients with cyanotic heart disease. To be extracted from an article, the reported risk factor was required to be calculated on at least 75% of the PICU sample. In case only median FSIQ was reported, we calculated the mean [22] and standard deviation (SD) [23]. In case SD of FSIQ was not provided, we used the normative SD (i.e. 15). Two reviewers independently extracted data. Any disagreements were solved through discussion or by consulting a third author.

### Study quality

Study quality was assessed using the Newcastle–Ottawa Scale for cohort studies [24]. According to the manual, the scale was adapted to fit the goal of this study (see Additional file 1 for more information [24–26]). All included studies were independently rated by two authors and disagreements were solved through discussion or by consulting a third author.

### Statistical analysis

Statistical analyses were performed using Comprehensive Meta-Analysis Software version 3.0.[27] For each individual study, FSIQ differences between PICU survivors and either healthy children or normative data were expressed in terms of standardized mean difference scores (Cohen’s *d*) and used as effect size. In case no healthy control group was included in a study, we used normative data for FSIQ (i.e. mean 100 and SD 15) assuming the same sample size as the PICU sample. Cohen’s *d* values of 0.2, 0.5 and 0.8, were used to define thresholds for small, medium and large effect sizes, respectively [28].

We calculated a meta-analytic effect size for FSIQ based on all included PICU samples. If a study reported on multiple patient samples separately, one combined effect size was calculated across patient samples before meta-analytic aggregation across studies [29]. In addition, we calculated meta-analytic effect size for a number of PICU subgroup based on the reported indications for PICU admission in the included studies. The available studies allowed to distinguish subgroups of children admitted for: [1] respiratory and/or circulatory insufficiency necessitating ECMO, [2] circulatory insufficiency necessitating CPR [3] traumatic brain injury [4] sepsis and/or meningoencephalitis [5] cardiac surgery [6] heart- or heart–lung transplantation and [7] miscellaneous PICU admission indications. The effect size of each study was weighted by the inverse of its variance to account for sample size and measurement error. Random-effects meta-analysis was performed, recognizing sources of inter-sample variance. Dispersion in effect sizes was quantified using *I*^*2*^, discriminating between mild (*I*^*2*^ < 30), moderate (*I*^*2*^ = 30–50) and strong heterogeneity (*I*^*2*^ > 30) [30]. Indications for publication bias were evaluated using funnel plots and the Egger’s test of asymmetry [31], while the robustness of the meta-analytic effect sizes was calculated by the fail-safe N value, where values > 5* k* + 10 were considered robust [32].

In order to determine risk factors for poor intelligence outcome, aggregated effect sizes of PICU subgroups were compared by Cochran’s Q to study whether subgroups differ in the risk for poor intelligence outcome. Subsequently, random-effects meta-regression analyses were performed to quantify the association of each of the demographic and clinical risk factors and the study’s effect sizes for FSIQ. These analyses were performed in the total sample of selected studies and in each PICU subgroup. Meta-regression analyses with < 10 observations were omitted [33].

## Results

**Fig. 1:**
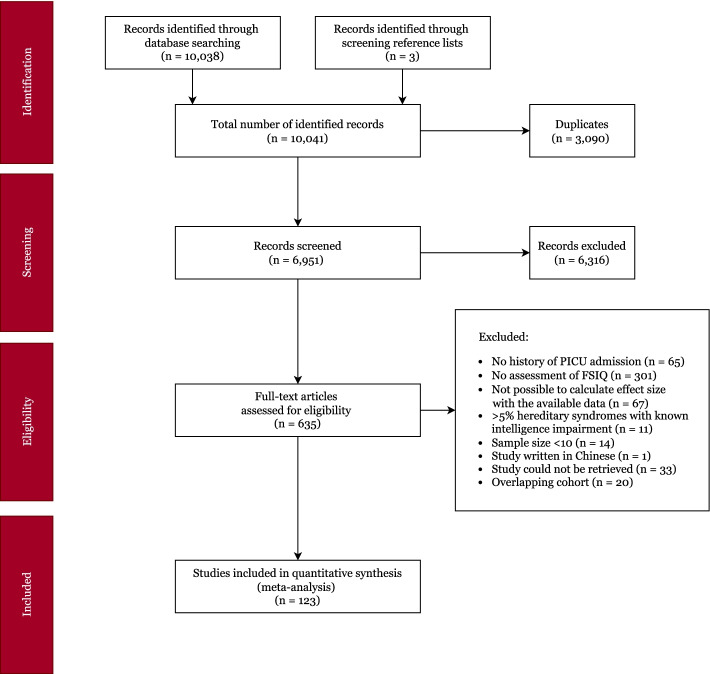
PRISMA flowchart of the study selection procedure. Note: FSIQ = full-scale intelligence quotient, PICU = pediatric intensive care unit

**Fig. 2:**
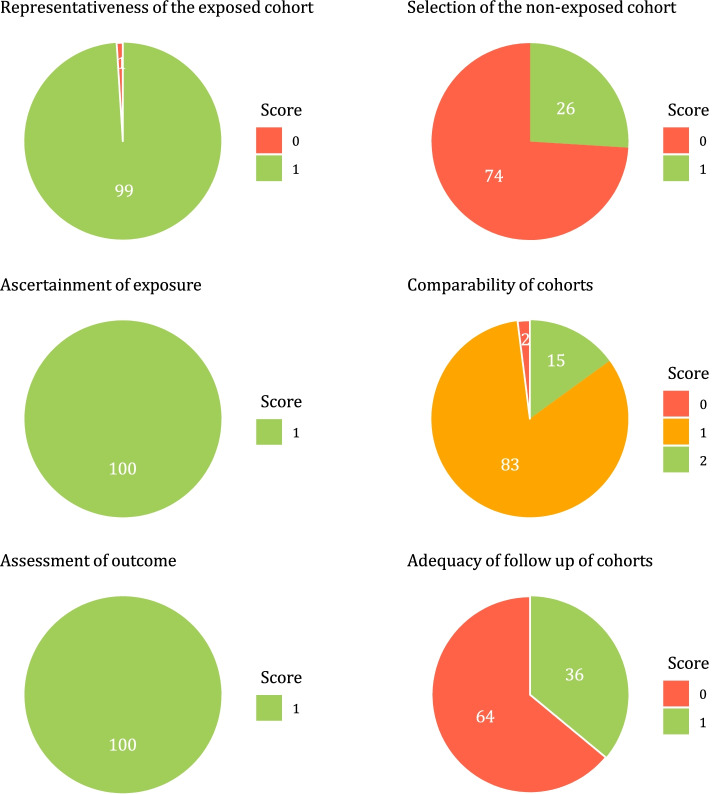
Overview of Quality assessment results. Note: Labels display percentages. Higher scores indicate higher study quality. See Supplemental Information for more information on the Newcastle Ottawa scale

Figure 1 shows the study selection process. Full-text examination revealed 123 eligible studies, published between 1973 and 2021 and comprising 8,119 PICU survivors. Thirty-three studies contained a healthy control group, representing 1,757 healthy control children. Mean year of PICU admission was 2000 (range 1972–2016, *k* = 99), mean percentage of boys was 59.1% (range 27.0–80.8%, *k* = 103), mean gestational age was 39.2 weeks (range 35.7–40.6 weeks, *k* = 46), mean age at PICU admission was 22.4 months (range 0.0–159.6, *k* = 103), mean time to follow-up was 68.8 months (range 0–231.6, *k* = 107) and mean age at follow-up was 92.8 months (range 30.1–307.2 months, *k* = 112). Additional file 1: Table S1 and S2 provide details of all included studies. Inter-rater agreement was 77.4% for study eligibility and 93.3% for quality assessment. The results of quality assessment at the group level are displayed in Fig. 2.

### FSIQ

**Fig. 3:**
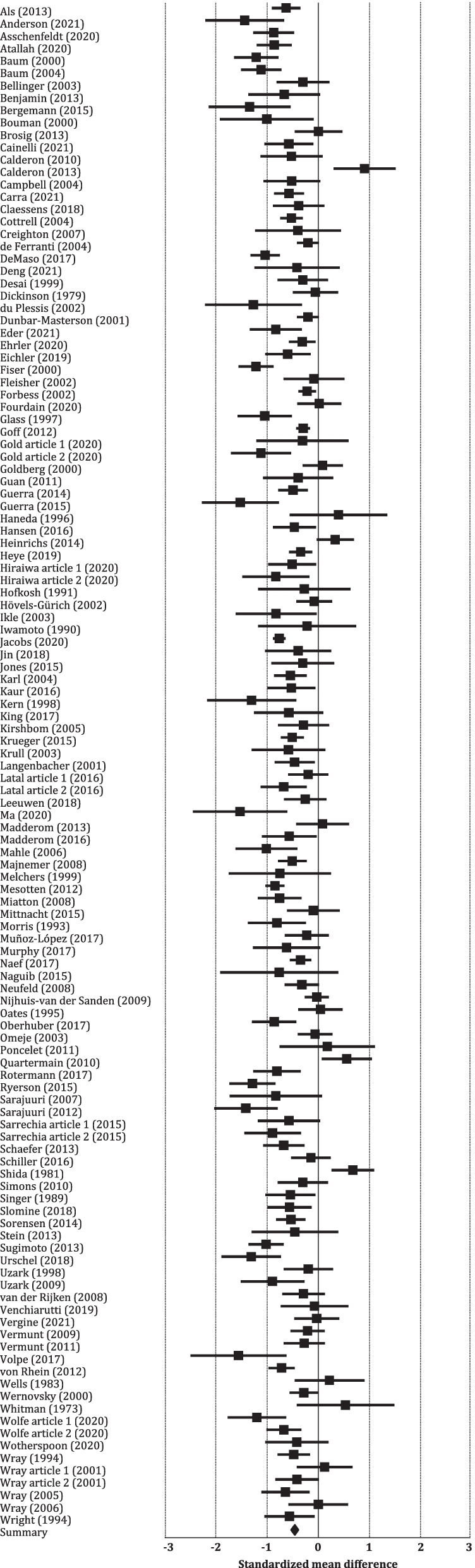
Forest plot showing standardized mean differences and accompanying 95% CI of studies comparing FSIQ of PICU survivors to healthy controls or normative data

The results of meta-analysis aggregating the results of all 123 studies comparing PICU samples to either healthy controls or normative data (further referred to as controls) are displayed in Fig. 3. The results reveal a small-sized aggregated effect size of *d* -0.47 (95% CI -0.55 to -0.40, *p* < 0.001), translating into a FSIQ impairment of on average 7.1 points in PICU survivors. There was strong heterogeneity in the individual study’s effect sizes (*I*^*2*^ = 71.20; *p* < 0.001).

### Risk factors

To study possible sources of the heterogeneity in the individual study’s effect sizes, we analysed subgroups of children based on the reported reasons for PICU admission. All subgroups had significantly lower FSIQ scores than controls (Table 1 and Additional files 2, 3, 4, 5, 6, 7, 8: Figures S1-7). The role of PICU subgroup as risk factor was determined by comparison of the aggregated effect sizes for FSIQ between subgroups. The results indicate that children admitted after heart- or heart–lung transplantation had significantly greater FSIQ impairment (*d* = -0.80) compared to children admitted after cardiac surgery (*d* = -0.38, Q = 9.48, *p* = 0.002) and compared to children admitted for sepsis and/or meningoencephalitis (*d* = -0.39, Q = 5.85, *p* = 0.02). Other comparisons between subgroups revealed no significant differences.

**Table 1:** Meta-analytic findings and results of the publication bias analyses for PICU subgroups

Subgroup	*k*	Cohen’s *d*	95% CI	Difference in IQ-points	Egger test of asymmetry (*p*-value)	Fail-safe *N*
Respiratory and/or circulatory insufficiency necessitating ECMO	10	-0.52 **	-0.81, -0.22	-7.76	.10	88
Circulatory insufficiency necessitating CPR	3	-0.88 **	-1.39, -0.37	-13.23	.13	19
Traumatic brain injury	3	-0.86 **	-1.48, -0.24	-12.84	.48	8
Sepsis and/or meningoencephalitis^a^	5	-0.39 ***	-0.61, -0.18	-5.88	.43	15
Cardiac surgery	80	-0.38 ***	-0.46, -0.30	-5.75	.59	5077
Heart- or heart–lung transplantation	14	-0.80 ***	-1.06, -0.55	-12.06	.44	368
Miscellaneous PICU admission indications	14	-0.55 ***	-0.75, -0.34	-8.19	.07	426

*k* = number of samples; CPR = cardio-pulmonary resuscitation; ECMO = extra-corporeal membrane oxygenation; PICU = pediatric intensive care unit. Difference in IQ-points compared to healthy controls or normative data. *p < .05. **p < .01. ***p < .001

aThis subgroup contains one sample with non-neurological sepsis

**Fig. 4:**
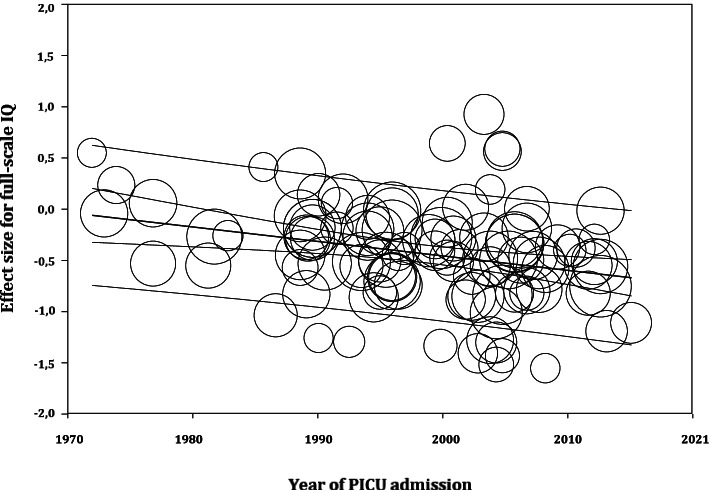
Association between year of PICU admission and study’s individual effect sizes for FSIQ. Note: Plotting characters are proportional to the study weight

The relation between demographic and clinical risk factors and the study’s individual effect sizes for FSIQ was investigated using meta-regression in the total sample (Table 2). Later year of PICU admission was significantly related to greater FSIQ impairment (R^2^ = 21%, see also Fig. 4), indicating that intelligence outcome of PICU survivors dropped with an average of 2.1 IQ-points every decade between 1972–2016. Furthermore, sex was significantly related to FSIQ (R^2^ = 5%). This finding indicates that one percentage increase in the percentage of boys in a study was related to an increase of on average 0.1 IQ-points (range studied 27.0–80.8%). In addition, longer PICU staywas significantly related to greater FSIQ impairment (R^2^ = 2%), indicating that intelligence outcome of PICU survivors dropped with an average of 1.5 IQ-points every additional week of PICU stay (range studied 0.3–35.4 days). Lower rate of survivors (range studied 38.2–100%) was significantly related to greater FSIQ impairment (R^2^ = 11%), which suggests that survivors in samples with higher mortality have poorer intelligence outcome. Last, higher study quality, as rated on the Newcastle–Ottawa Scale (range 3–7), was significantly related to greater FSIQ impairment (R^2^ = 7%). No other significant relationships were observed. Of note, no multivariate meta-regression analysis was conducted, because of the low number of studies (*k* = 29) that reported all risk factors that were found significantly related to FSIQ in the univariate meta-regression analysis, which would lead to biased and underpowered analysis.

**Table 2:** Results of univariate meta-regression analyses of demographic and clinical risk factors for FSIQ impairment

**Risk factors**	***k***	**Beta**	**95% CI**	**R**^2^ **(%)**	**Range studied**
Year of PICU admission	104	-0.014 ***	-0.021, -0.007	21	1972–2016
Sex (% boys)	107	0.007 *	0.001, 0.014	5	27.0–80.8
Gestational age (weeks)	49	-0.069	-0.188, 0.051	0	35.7–40.6
Age at PICU admission (months)	107	0.000	-0.002, 0.002	1	0.0–159.6
Mechanical ventilation (days)	21	-0.011	-0.030, 0.007	0	0.0–41.5
PICU stay (days)	36	-0.014 *	-0.027, -0.002	2	0.3–35.4
Resuscitation (%)	22	-0.005	-0.011, 0.001	2	0.0–100
ECMO (%)	28	-0.002	-0.005, 0.002	0	0.0–100
Rate of survivors (%)	56	0.014 ***	0.006, 0.022	11	38.2–100
Age at follow-up (months)	117	0.000	-0.001, 0.001	0	30.1–307.2
Time to follow-up (months)	110	-0.000	-0.002, 0.001	0	0.1–231.6
Study quality	129	-0.109 *	-0.198, -0.020	7	3–7

*k* = number of samples; ECMO = extra-corporeal membrane oxygenation; PICU = pediatric intensive care unit; Study quality was assessed by the Newcastle Ottawa Scale for cohort studies, revised to a maximum of 7 points. Unstandardized Beta’s are reported. *p < .05. **p < .01. ***p < .001

Meta-regression in PICU subgroups was possible (i.e. > 10 observations) in the subgroups of children with respiratory and/or circulatory insufficiency necessitating ECMO, cardiac surgery and heart- or heart–lung transplantation (Additional file 1: Table S3). Among children admitted after cardiac surgery, later year of PICU admission (range 1972–2013), lower percentage of boys (range 30.3–79.4%) and higher study quality (range 3–7), were related to greater FSIQ impairment (R^2^ = 12%, 6% and 9%, respectively). Among children admitted after heart- or heart–lung transplantation, later year of PICU admission (range 1989–2016), younger age at PICU admission (range 1.6–118.4 months) and younger age at follow-up (range 40.7–166.8 months) were related to greater FSIQ impairment (R^2^ = 65%, 74% and 68%, respectively). None of the other risk factors were related to FSIQ impairment in any of the subgroups.

### Publication bias

Inspection of the funnel plot in the total sample did not suggest publication bias (Additional file 9: Figure S8), Egger’s test of asymmetry was not significant (*p* = 0.50) and the fail-safe N (*N* = 7,559) indicated that the obtained effect size was robust. Similar results were obtained in the PICU subgroups, with the exception that the fail-safe N values did not support the robustness of the effect sizes obtained in the subgroups of children admitted for circulatory insufficiency necessitating CPR, traumatic brain injury and children with sepsis and/or meningoencephalitis (Table 1).

### Use of normative data in uncontrolled studies

We explored the validity of using normative data for the calculation of effect sizes in studies not including a healthy control group. Hence, we calculated effect sizes for studies including a healthy control group (*k* = 31) with two approaches: [1] using data of the healthy control group and [2] using normative data (i.e. mean 100 and SD 15). Comparisons between the effect sizes retrieved with these two methods revealed a significant difference (Q = 39.5, *p* < 0.001), with the approach using healthy control group data resulting in a larger aggregated effect size (healthy control group: *d* = -0.62, 95% CI -0.74 to -0.51, *p* < 0.001 vs. normative data: *d* = -0.00, 95% CI -0.16 to 0.16, *p* = 0.99). This finding was replicated when selecting only those studies with a healthy control group that also tested and confirmed comparability of the PICU and healthy control groups in terms of sex, age and socioeconomic status (most often defined by parental level of education; *k* = 14; Q = 35.5, *p* < 0.001).. These findings indicate that the use of normative data yields conservative estimates of FSIQ impairment in PICU survivors.

## Discussion

This meta-analysis and meta-regression aimed to [1] quantify intelligence outcome after PICU admission; and [2] explore risk factors for poor intelligence outcome. Based on 123 studies including 8,119 PICU survivors and 1,757 healthy control children, our results demonstrate 0.47 SD lower intelligence scores in PICU survivors compared to controls (healthy control children or normative data), corresponding to an average difference of 7.1 IQ-points. Accordingly, the prevalence of children with intellectual disability (FSIQ < 2 SD [34]) is expected to be threefold higher in PICU survivors (6.4%) than in the general population (2.3%). Intelligence reflects the ability to efficiently process information for goal-directed behavior and is known to be related to physical and mental health [9, 10, 35], academic achievement [11], socioeconomic success [12] and survival to old age [35]. Even a small difference in intelligence can affect profound effects on life chances [10]. These findings highlight the relevance of intelligence outcome and stress the relevance of structured neurocognitive follow-up of PICU survivors.

The results of our study show intelligence impairment across all PICU subgroups investigated, with effect sizes ranging between -0.38 and -0.88 SD. Children admitted after heart- or heart–lung transplantation had significantly greater intelligence impairment (-0.80 SD) compared to children admitted after cardiac surgery (-0.38 SD), and compared to children admitted for sepsis and/or meningoencephalitis (-0.39 SD). This finding may reflect the greater disease severity, greater intensity of PICU treatments, and/or greater intensity of surgical treatment(s) of children admitted after heart- or heart–lung transplantation. The results on the PICU subgroups in the current study are in line with earlier literature overviews [36–42] and extend these findings by the unique focus on children admitted to the PICU and by providing comprehensive meta-analytic quantification of intelligence impairment.

Meta-regression allowed to study a broad range of demographic and clinical risk factors for intelligence outcome. The results showed that later year of PICU admission (range studied 1972 – 2016) was related to greater intelligence impairment (R^2^ = 21%). These findings may reflect the increasing medical attainments that have not only led to increased survival rates of children admitted to the PICU, but also to increasing morbidity rates in those surviving [1, 2, 43]. This hypothesis does not find direct support by the contrasting observation that lower rate of survivors (range studied: 38.2–100%) was related to greater intelligence impairment (R^2^ = 11%). However, differences between survival rate in this analysis may not only reflect potential trends over time, but also differences in the severity of critical illness between conditions that may influence intelligence outcome. In line with this idea, results showed that longer PICU stay (range studied 0.3–35.4 days) was related to greater intelligence impairment (R^2^ = 2%). This finding may reflect the greater disease severity and/or the greater intensity of PICU treatments of children with longer PICU stay, which may have affected their long-term neurocognitive outcome. Our findings are corroborated by a recent systematic review which also showed that length of PICU stay was related to poorer neurocognitive functioning at discharge [44]. Of note, our current findings indicate that boys had on average better intelligence outcome than girls (R^2^ = 5%), i.e. every 10 percentage points increase in the amount of boys was related to an increase of on average 1 IQ-point (range studied 27.0–80.8%). No evidence was found for a confounding effect, i.e. girls were not overrepresented in any of the PICU subgroups. The mechanisms underlying sex differences with respect to prevalence and outcome of several neurological conditions are currently not well understood [45]. Sex differences exist in, amongst others, different states in neuroinflammation [45] and (hormonal) reaction to stress [46–48]. These sex differences may possibly lead to differences in neurocognitive development of PICU survivors. Understanding the mechanisms behind sex differences could help develop more targeted therapy. At last, meta-regression showed that higher study quality was related to greater intelligence impairment (R^2^ = 7%). This aligns with the findings of our additional analysis, which showed that the use of the normative mean instead of control group data provides conservative estimates of intelligence impairment.

Regarding subgroups, the meta-regression findings of the total sample were replicated in the subgroup of children admitted after cardiac surgery, with the exception that length of PICU stay was not significantly related to intelligence in this subgroup. Interestingly, longer CPB duration was not related to greater intelligence impairment. This finding contrasts with existing literature from adults showing that CPB duration is related to length of intensive care unit and hospital stay and in-hospital mortality [49]. Taken together, the potential relation between CPB duration and complication risk may not translate into intelligence outcome in children. The results of this study further show that later year of PICU admission was also related to greater intelligence impairment in children with heart- or heart–lung transplantation. In addition, results indicate that younger age at PICU admission was related to greater intelligence impairment in this subgroup. One possible explanation for this finding may be that the main reasons for heart transplantation differ with age (i.e. < 1 year congenital heart disease, > 1 year cardiomyopathy)[50] and congenital heart disease may impact brain development already before birth [51]. We also found that older age at follow-up was associated with smaller intelligence impairment in this subgroup, suggesting that intelligence outcome after heart- or heart–lung transplantation may improve over time.

Intelligence impairment in PICU survivors may be caused by complex interaction between factors related to premorbid functioning [52], underlying disease [53], critical illness [17] and intensive care treatment [54], which influence pathophysiological mechanisms involving (a combination of) hypoxia, metabolic derangements such as glucose dysregulation, ischemia, inflammation, hypotension, delirium and potential negative effects of sedation [13–15]. We can only speculate about the specific active (combination) of underlying mechanisms that fuel intelligence impairment in critically ill children admitted to the PICU, which is also likely to differ between subgroups. Nevertheless, our study indicates the need for appropriate prospective studies that provide insight into the potential contribution of pathophysiological mechanisms to intelligence outcome. Such studies may expose potential targets for treatment innovations that may benefit outcome of PICU survivors.

One limitation of this study is that a limited number of possible risk factors was assessed in the included studies (e.g. none of the studies assessed medical history prior to or after PICU admission) and the number of missing data for demographic and clinical potential risk factors was considerable. This reduced the power to identify risk factors (particularly in subgroups). Nevertheless, the available data did allow us to study a broad range of risk factors in the total sample of studies. Furthermore, the current study is limited by the availability of studies into intelligence outcome after PICU admission, with the available studies likely not being fully representative of the typical PICU population in terms of reasons for admission. Our study shows that a substantial number of studies is published mainly on the subgroup of children admitted after cardiac surgery, while other subgroups are less well studied or not at all. For example, we were not able to identify studies including children with respiratory insufficiency necessitating mechanical ventilation or renal insufficiency necessitating renal replacement therapy in our broad and extensive systematic search, while these are important indications for PICU admission [1, 2] and concerns about neurocognitive development of these PICU subgroups exist [4]. This limits the generalizability of our results to the PICU population as a whole and underscores the need for more follow-up studies on these populations. A strength of our study is that with our broad and extensive systematic search we included a considerable number of studies and we were able to aggregate all existing data on intelligence outcome of PICU survivors, to systematically report on subgroups and to comprehensively study risk factors for intelligence impairment. Second, we showed that the use of normative data might underestimate the estimates of intelligence impairment in PICU survivors. Critical appraisal of the role of control data used is important, as normative data are frequently used in research and this may considerably influence the results and conclusions of studies.

## Conclusion

In this meta-analysis, robust evidence was found for a risk of intelligence impairment in PICU survivors, applying to a wide range of PICU subgroups. The results further indicate worsening intelligence outcome in the PICU populations over the years (between 1972–2016), potentially reflecting the increasing percentage of children surviving PICU admission with morbidity. In addition, the results indicate that longer length of PICU stay, female sex and lower rate of survivors negatively influence intelligence outcome after PICU admission. The findings of this meta-analysis warrant the need for structured neurocognitive follow-up of PICU survivors.

## Supplementary Information

**Additional file 1. **Table S1-S3, additional information on the assessment of the study quality, and the search strategy. Table S1 - Details on PICU groups studied in the included studies, Table S2 - Study characteristics and FSIQ differences (Cohen’s d) between PICU survivors and controls, Table S3 - Results of univariate meta-regression analyses of risk factors for FSIQ impairment in PICU subgroups. 

**Additional file 2. **Figure S1 - Forest plot showing standardized mean differences and accompanying 95% CI of studies reporting on the subgroup Respiratory and/or circulatory insufficiency necessitating ECMO, comparing FSIQ of PICU survivors to healthy controls or normative data.

**Additional file 3.** Figure S2 - Forest plot showing standardized mean differences and accompanying 95% CI of studies reporting on the subgroup Circulatory insufficiency necessitating CPR, comparing FSIQ of PICU survivors to healthy controls or normative data.

**Additional file 4. **Figure S3 - Forest plot showing standardized mean differences and accompanying 95% CI of studies reporting on the subgroup Traumatic brain injury, comparing FSIQ of PICU survivors to healthy controls or normative data.

**Additional file 5.** Figure S4 - Forest plot showing standardized mean differences and accompanying 95% CI of studies reporting on the subgroup Sepsis and/or meningoencephalitis, comparing FSIQ of PICU survivors to healthy controls or normative data.

**Additional file 6.** Figure S5 - Forest plot showing standardized mean differences and accompanying 95% CI of studies reporting on the subgroup Cardiac surgery, comparing FSIQ of PICU survivors to healthy controls or normative data.

**Additional file 7.** Figure S6 - Forest plot showing standardized mean differences and accompanying 95% CI of studies reporting on the subgroup Heart- or heart-lung transplantation, comparing FSIQ of PICU survivors to healthy controls or normative data.

**Additional file 8.** Figure S7 - Forest plot showing standardized mean differences and accompanying 95% CI of studies reporting on the subgroup Miscellaneous PICU admission indications, comparing FSIQ of PICU survivors to healthy controls or normative data.

**Additional file 9.** Figure S8 - Funnel plot of the study’s individual effect sizes for FSIQ plotted against its standard error.

**Additional file 10.** References of the studies included in the meta-analysis.

## Data Availability

All data generated or analysed during this study are included in this published article in its supplementary information files.

## Abbreviations

CPB: Cardiopulmonary bypass
CPR: Cardiopulmonary resuscitation
DHCA: Deep-hypothermic circulatory arrest
ECMO: Extra-corporeal membrane oxygenation
FSIQ: Full-scale intelligence quotient
PICU: Pediatric intensive care unit
