# Supplementary material for: Intelligence outcome of pediatric intensive care unit survivors: a systematic meta-analysis and meta-regression
Source: BMC Med. 2022 Jun 1;20:198. doi: 10.1186/s12916-022-02390-5 (PMC9158152; doi:10.1186/s12916-022-02390-5)
Supplement: Supplementary file 1 — Additional file 1. Table S1-S3, additional information on the assessment of the study quality, and the search strategy. Table S1 - Details on PICU groups studied in the included studies, Table S2 - Study characteristics and FSIQ differences (Cohen’s d) between PICU survivors and controls, Table S3 - Results of univariate meta-regression analyses of risk factors for FSIQ impairment in PICU subgroups. [file 12916_2022_2390_MOESM1_ESM.docx]

**Intelligence Outcome of Pediatric Intensive Care Unit Survivors:
A systematic meta-analysis and meta-regression**

**Additional file 1**

| **Table S1.** Details on PICU groups studied in the included studies. | | | | | | | | | | | | | | | | |  |
| --- | --- | --- | --- | --- | --- | --- | --- | --- | --- | --- | --- | --- | --- | --- | --- | --- | --- |
| **Study name** | **Subgroup** | **Country** | **Sex  (% boys)** | **Gestational age (weeks)** | **Age at follow-up (months)** | **Time to follow-up (months)** | **Age at PICU admission (months)** | **PICU stay (days)** | **Mechanical ventilation (days)** | **Resusci-tation (%)** | **ECMO (%)** | **Year at PICU (mean)** | **CPB (%)** | **CPB duration (minutes)** | **DHCA (%)** | **Cyanotic heart disease (%)** | **Survival rate (%)** |
| Als (2013) | sepsis and/or meningoencephalitis | UK | NA | NA | NA | NA | 120.83 | 4.10 | NA | NA | NA | 2008 | NA | NA | NA | NA | NA |
| Als (2013) | miscellaneous | UK | NA | NA | NA | NA | 115.75 | 1.18 | NA | NA | NA | 2008 | NA | NA | NA | NA | NA |
| Anderson (2021) | heart- or heart-lung tx | Canada | 54.3 | NA | NA | NA | NA | NA | NA | NA | NA | 2005 | 100 | 215.8 | 24.29 | NA | 80 |
| Asschenfeldt (2020) | cardiac surgery | Denmark | 30.3 | NA | 307.2 | 231.6 | 93.6 | 1.28 | NA | NA | NA | 1995 | 100 | 51.3 | 0 | 0 | NA |
| Atallah (2020) | cardiac surgery | Canada | 70.60 | 39.0 | 56.6 | 56.3 | 0.3 | 25.3 | 21.4 | 5.9 | 1.5 | 2003 | 100 | 113.1 | 100 | 100 | 58 |
| Baum (2000) | heart- or heart-lung tx | USA | NA | NA | 84 | 82.3 | 1.7 | NA | NA | NA | NA | NA | 100 | NA | 100 | NA | 73 |
| Baum (2004) | heart- or heart-lung tx | USA | 58 | 39.4 | 76.8 | 75.16 | 1.64 | NA | NA | NA | NA | NA | 100 | NA | 100 | 90.9 | NA |
| Bellinger (2003) | cardiac surgery | USA | 76.32 | 39.74 | 98.37 | 98.04 | 0.33 | NA | NA | NA | NA | 1990 | 100 | 108.3 | 100 | 100 | 96 |
| Benjamin (2013) | miscellaneous | USA | 68.75 | 38.5 | 62.8 | 62.3 | 0.5 | NA | 33.33 | NA | 25 | 2003 | NA | NA | NA | NA | 63 |
| Bergemann (2015) | cardiac surgery | Germany | 60.09 | NA | 95.83 | 95.52 | 0.32 | NA | NA | 12.27 | NA | 2000 | 100 | 88.6 | 26.32 | 100 | NA |
| Bouman (2000) | miscellaneous | The Netherlands | 54.55 | NA | 124.83 | 124.8 | 0.03 | NA | 35.77 | NA | 0 | NA | NA | NA | NA | NA | NA |
| Brosig (2013) | cardiac surgery | USA | 64.70 | 38.20 | 60 | 59.79 | 0.21 | NA | NA | NA | 11.8 | 2002 | 100 | 328.5 | 100 | 100 | 88 |
| Cainelli (2021) | cardiac surgery | Italy | 57 | NA | 87.12 | 79.92 | 15.20 | 6.60 | NA | NA | NA | NA | 100 | 127 | NA | NA | NA |
| Calderon (2010) | cardiac surgery | France | 66.67 | NA | 88 | 87.5 | 0.5 | NA | NA | 0 | NA | 2001 | 100 | NA | NA | 100 | NA |
| Calderon (2013) | cardiac surgery | France | 66.67 | 38.91 | 65.75 | 65.52 | 0.23 | 6.55 | NA | NA | NA | 2004 | 100 | 130.1 | 0 | 100 | NA |
| Campbell (2004) | TBI | Canada | NA | 40 | NA | 27 | NA | NA | NA | NA | NA | 1995 |  | NA | NA | NA | 87 |
| Carra (2021) | cardiac surgery | Belgium | 64 | NA | 30.1 | 24 | 6.1 | 7 | NA | 0 | NA | 2013 | 89 | 77 | 4 | 62 | 95.9 |
| Claessens (2018) | cardiac surgery | The Netherlands | 79.41 | 39.28 | 71 | 70.66 | 0.34 | NA | NA | NA | NA | 2010 | 100 | NA | NA | 58.82 | 92 |
| Cottrell (2004) | cardiac surgery | USA | NA | NA | 48 | 45 | 3 | NA | NA | NA | NA | 1994 | 100 | NA | 100 | 100 | NA |
| Creighton (2007) | cardiac surgery | Canada | 68.85 | NA | 60 | 59.31 | 0.69 | NA | NA | NA | NA | 1997 | 100 | 75.8 | 75.42 | 100 | 78 |
| de Ferranti (2004) | cardiac surgery | USA | 75 | 40.04 | 96 | 95.73 | 0.27 | NA | NA | NA | NA | 1990 | 100 | NA | 50.88 | 100 | NA |
| DeMaso (2017) | cardiac surgery | USA | 71.43 | 39.3 | 168 | NA | NA | NA | NA | NA | NA | NA | 100 | NA | NA | 100 | NA |
| Deng (2021) | sepsis and/or meningoencephalitis | Australia | 54.5 | NA | 66.61 | 47 | 19.59 | 3 | NA | NA | NA | 2011 | NA | NA | NA | NA | NA |
| Desai (1999) | ECMO | USA | NA | 40.55 | 63.55 | 63.47 | 0.08 | NA | NA | 19.19 | 100 | 1989 | NA | NA | NA | NA | NA |
| Dickinson (1979) | cardiac surgery | UK | NA | NA | 53.42 | 42.54 | 10.88 | NA | NA | NA | NA | 1973 | 100 | NA | 100 | 65.79 | 64 |
| du Plessis (2002) | cardiac surgery | USA | NA | NA | 81.6 | 76.8 | 4.8 | NA | NA | NA | NA | 1990 | 100 | 83.5 | 70 | 70 | 92 |
| Dunbar-Masterson (2001) | cardiac surgery | USA | 76 | 39.8 | 100.15 | 99.88 | 0.27 | 6.7 | NA | NA | NA | 1990 | 100 | 108 | 100 | 100 | 96 |
| Eder (2021) | miscellaneous | Germany | 54 | NA | 126 | 70.3 | 55.7 | 19.9 | NA | 0.0 | NA | 2012 | NA | NA | NA | NA | NA |
| Ehrler (2020) | cardiac surgery | Switzerland | 65 | NA | 122.4 | NA | NA | NA | NA | NA | NA | 2006 | 100 | NA | NA | 36 | 91 |
| Eichler (2019) | cardiac surgery | Germany | 41.03 | 39 | 87.6 | 74.76 | 12.84 | NA | NA | NA | NA | 2009 | 100 | NA | NA | 0 | 99 |
| Fiser (2000) | miscellaneous | USA | 62 | NA | 147 | 0 | 147 | NA | NA | NA | NA | NA | NA | NA | NA | NA | NA |
| Fleisher (2002) | cardiac surgery | USA | 73 | 40 | 79.19 | 72 | 7.19 | NA | NA | NA | NA | 1992 | 100 | 102 | NA | NA | NA |
| Fleisher (2002) | heart- or heart-lung tx | USA | 44 | 40 | 79.52 | 72 | 7.52 | NA | 6.38 | NA | NA | 1992 | 100 | 93 | 100 | NA | NA |
| Forbess (2002) | cardiac surgery | USA | 56.79 | NA | 67.72 | 58.63 | 9.09 | 5 | NA | NA | NA | 1996 | 82 | 133 | 40 | NA | NA |
| Fourdain (2020) | cardiac surgery | Canada | 46.3 | 38.7 | 44.1 | 43.51 | 0.59 | 6.5 | NA | NA | NA | NA | 67.5 | 161.9 | NA | 57.5 | NA |
| Glass (1997) | ECMO | USA | 69.16 | 38.91 | 60.50 | 60 | 0.50 | NA | 0.10 | NA | 100 | 1987 | NA | NA | NA | NA | NA |
| Goff (2012) | cardiac surgery | USA | 56.61 | 38 | 54 | 53.61 | 0.39 | NA | NA | NA | NA | 2000 | 100 | 49 | 57 | NA | 87 |
| Gold article 1 (2020) | miscellaneous | Canada | 42.90 | NA | 143.16 | 13.80 | 129.36 | NA | NA | NA | NA | 2013 | NA | NA | NA | NA | NA |
| Gold article 2 (2020) | heart- or heart-lung tx | Canada | 28.0 | NA | 79.65 | 69.88 | 9.77 | 31.1 | NA | NA | 8 | 2016 | 100 | NA | NA | NA | NA |
| Goldberg (2000) | cardiac surgery | USA | 66.67 | NA | 57.6 | 20 | 37.6 | NA | NA | 14.6 | NA | 1992 | 100 | NA | 68.63 | 100 | NA |
| Guan (2011) | cardiac surgery | China | 68.97 | NA | NA | NA | NA | NA | NA | NA | NA | 2002 | 100 | 73.6 | 0 | 0 | NA |
| Guerra (2014) | cardiac surgery | Canada | 61.54 | 38.9 | 54 | 53.62 | 0.38 | 16.8 | 9.9 | 4 | 3 | 2004 | 100 | NA | NA | 100 | 86 |
| Guerra (2015) | CPR | Canada | 50.91 | NA | 52 | NA | NA | NA | NA | 100 | 100 | 2005 | NA | NA | NA | NA | 38.2 |
| Haneda (1996) | cardiac surgery | Japan | NA | NA | NA | 0.70 | NA | NA | NA | NA | NA | 1986 | 100 | NA | NA | 68.29 | NA |
| Hansen (2016) | cardiac surgery | Germany | 65.12 | 39.7 | 55.5 | 55.27 | 0.23 | NA | NA | NA | NA | 2008 | 100 | 385 | NA | 100 | 80 |
| Heinrichs (2014) | cardiac surgery | Germany | 78.3 | NA | 202.8 | 202.57 | 0.23 | NA | NA | 6.7 | NA | 1989 | 100 | 61.7 | 100 | 100 | 93 |
| Heye (2019) | cardiac surgery | Switzerland | 63.64 | 39.47 | 75.55 | 73.98 | 1.57 | 16.44 | NA | NA | NA | 2006 | 100 | 196 | 17.48 | 71 | 96 |
| Hiraiwa article 1 (2020) | cardiac surgery | Japan | 54.3 | 39.2 | 89.5 | NA | NA | NA | NA | 8.6 | 0 | 2007 | 100 | 178 | 5.7 | 100 | 89 |
| Hiraiwa article 2 (2020) | cardiac surgery | Japan | 51.9 | 39.2 | 113.1 | NA | NA | NA | NA | NA | 0 | 2006 | 100 | NA | NA | 100 | 95.7 |
| Hofkosh (1991) | ECMO | USA | 69.47 | NA | 64.14 | 63.64 | 0.50 | NA | NA | NA | 100 | 1983 | NA | NA | NA | NA | NA |
| Hövels-Gürich (2002) | cardiac surgery | Germany | 76.67 | NA | 126 | 125.77 | 0.23 | NA | NA | 6.7 | NA | 1989 | 100 | 63.4 | 100 | 100 | 91 |
| Ikle (2003) | heart- or heart-lung tx | USA | 80.77 | NA | NA | NA | NA | NA | NA | NA | NA | 1995 | 100 | NA | 100 | 100 | 70 |
| Iwamoto (1990) | cardiac surgery | Japan | NA | NA | 148 | 72 | 76 | NA | NA | NA | NA | NA | 100 | NA | 100 | 1.33 | NA |
| Jacobs (2020) | miscellaneous | Belgium, The Netherlands and Canada | 57.46 | NA | 87.6 | 48.0 | 39.6 | 7.8 | 5.0 | NA | NA | 2013 | NA | NA | NA | NA | 90.5 |
| Jin (2018) | cardiac surgery | China | 68.97 | NA | NA | NA | NA | NA | NA | NA | NA | 2011 | 100 | 73.6 | NA | 0 | NA |
| Jones (2015) | cardiac surgery | Australia | 50 | NA | 79.2 | 77.5 | 1.7 | NA | NA | NA | NA | 2000 | 100 | 155 | 25 | NA | NA |
| Karl (2004) | cardiac surgery | Australia | NA | 39.6 | 109.7 | 109.18 | 0.52 | 3 | NA | NA | NA | 1991 | 100 | 126 | 100 | 100 | NA |
| Kaur (2016) | sepsis and/or meningoencephalitis | India | 46 | NA | 93.6 | 6 | 87.6 | 4.9 | NA | NA | NA | 2013 | NA | NA | NA | NA | NA |
| Kern (1998) | cardiac surgery | USA | 58.33 | NA | 52.8 | 52.54 | 0.26 | NA | NA | NA | NA | 1993 | 100 | 188 | 100 | 100 | NA |
| King (2017) | cardiac surgery | USA | 70.6 | NA | 213.12 | 211.2 | 1.92 | NA | NA | NA | NA | NA | 100 | NA | NA | 100 | NA |
| Kirshbom (2005) | cardiac surgery | USA | 70 | 39 | 136.48 | 135.33 | 1.15 | 8 | NA | NA | 3.3 | 1989 | 100 | 71 | 100 | 100 | 81 |
| Krueger (2015) | cardiac surgery | Switzerland | 64.7 | 39.3 | 51.6 | 48.8 | 2.8 | 9.18 | 5.41 | NA | NA | 2006 | 100 | 165.3 | NA | 67.3 | 92 |
| Krull (2003) | miscellaneous | USA | 27 | NA | 81.6 | 66 | 15.6 | NA | NA | NA | NA | 1989 | NA | NA | NA | NA | NA |
| Langenbacher (2001) | ECMO | USA | 51.92 | 39 | 61 | 60.94 | 0.06 | NA | NA | NA | 100 | 1989 | NA | NA | NA | NA | 82 |
| Latal article 1 (2016) | cardiac surgery | Switzerland | 65 | 38.95 | 51.6 | 51.06 | 0.54 | 21.8 | 14.22 | 13.3 | 1.7 | 2007 | 100 | NA | NA | 70.3 | NA |
| Latal article 2 (2016) | cardiac surgery | Switzerland | 43.75 | NA | 166.36 | 146.54 | 19.82 | NA | NA | NA | NA | 1996 | 100 | 91 | NA | 50 | NA |
| Leeuwen (2018) | ECMO | The Netherlands | 54.29 | 40.29 | 96 | 95.50 | 0.50 | 27.70 | 17.61 | 8.57 | 100 | 2007 | NA | NA | NA | NA | 79 |
| Leeuwen (2018) | miscellaneous | The Netherlands | 60 | 38.54 | 96 | 95.5 | 0.5 | 22.78 | 10.36 | 0 | 0 | 2007 | NA | NA | NA | NA | 79 |
| Ma (2020) | cardiac surgery | China | 60.0 | NA | 120.12 | 94.92 | 25.20 | NA | NA | NA | NA | NA | 100 | 67.75 | NA | 100 | NA |
| Madderom (2013) | ECMO | The Netherlands | 62.5 | 39.5 | 96 | NA | NA | NA | 41.5 | NA | 100 | 2001 | NA | NA | NA | NA | 48.6 |
| Madderom (2013) | miscellaneous | The Netherlands | 42.11 | 37.63 | 96 | NA | NA | NA | 13.11 | NA | 0 | 2001 | NA | NA | NA | NA | 80 |
| Madderom (2016) | ECMO | The Netherlands | 47.06 | 39.89 | 144.07 | 144 | 0.07 | NA | 12.32 | NA | 100 | 1994 | NA | NA | NA | NA | 75 |
| Mahle (2006) | cardiac surgery | USA | 68.09 | 39.50 | 148.83 | 148.32 | 0.51 | NA | NA | NA | NA | NA | 100 | 90.5 | 100 | 100 | NA |
| Majnemer (2008) | cardiac surgery | Canada |  | 40 | 64.2 | 61.5 | 2.7 | 11.4 | NA | NA | NA | 1996 | 100 | 152.8 | 52 | 68.09 | 88 |
| Melchers (1999) | TBI | Germany | 61.35 | NA | NA | 12 | NA | NA | NA | NA | NA | NA | NA | NA | NA | NA | NA |
| Mesotten (2012) | miscellaneous | Belgium | 57.24 | NA | 72.86 | 47.22 | 25.64 | NA | NA | NA | NA | 2005 | NA | NA | NA | NA | 72.1 |
| Miatton (2008) | cardiac surgery | Belgium | 51.16 | NA | 104 | 98 | 6 | NA | NA | NA | NA | 1997 | 100 | NA | NA | 55.81 | NA |
| Mittnacht (2015) | cardiac surgery | Germany | 50 | 40 | 140.57 | NA | NA | NA | NA | NA | NA | 1994 | 100 | NA | NA | 60.7 | 98 |
| Morris (1993) | CPR | USA | 36 | NA | 67 | NA | NA | NA | NA | 100 | NA | NA | NA | NA | NA | NA | NA |
| Muñoz-López (2017) | cardiac surgery | UK | 70 | NA | 136.8 | 136.3 | 0.5 | NA | NA | NA | NA | 1995 | 100 | NA | NA | 100 | NA |
| Murphy (2017) | cardiac surgery | USA | 39 | NA | 193.2 | 190.13 | 3.07 | NA | NA | NA | NA | NA | 100 | 113.3 | NA | 100 | NA |
| Naef (2017) | cardiac surgery | Switzerland | 61.5 | 39.19 | 75.27 | 70.54 | 4.73 | 14.69 | NA | NA | NA | 2006 | 100 | 155.1 | NA | 63.3 | 93.2 |
| Naguib (2015) | cardiac surgery | USA | 70 | NA | 36.15 | 31.70 | 4.45 | 2.14 | NA | NA | NA | NA | 100 | 119.8 | NA | NA | NA |
| Neufeld (2008) | cardiac surgery | Canada | 64.62 | 39 | 58 | 57.64 | 0.36 | NA | 9 | 3 | 3 | 1999 | 100 | 137 | 50.77 | 100 | 98.5 |
| Nijhuis-van der Sanden (2009) | ECMO | The Netherlands | 58.39 | 39.5 | 62 | 61.5 | 0.5 | NA | NA | NA | 100 | 1996 | NA | NA | NA | NA | 78 |
| Oates (1995) | cardiac surgery | Australia | 59.52 | NA | 126.69 | 101.20 | 25.49 | NA | NA | NA | NA | 1977 | 100 | NA | 67.86 | 48.21 | NA |
| Oberhuber (2017) | cardiac surgery | Austria | 65.12 | NA | 123.6 | 123.1 | 0.5 | NA | NA | NA | NA | 2003 | 100 | NA | 100 | 100 | 57 |
| Omeje (2003) | cardiac surgery | Bratislava | 62 | NA | 66.13 | 63.05 | 3.08 | NA | NA | NA | NA | 1996 | NA | NA | NA | NA | NA |
| Poncelet (2011) | cardiac surgery | Belgium | NA | NA | NA | 48 | NA | NA | NA | NA | 2.2 | 2004 | 100 | NA | NA | NA | 95.6 |
| Quartermain (2010) | cardiac surgery | USA | 54 | NA | 147.6 | 6 | 141.6 | 2.1 | 0 | NA | NA | 2005 | 100 | 46 | NA | 0 | 100 |
| Quartermain (2010) | miscellaneous | USA | 74 | NA | 154.8 | 6 | 148.8 | 0.3 | 0 | NA | NA | 2005 | NA | NA | NA | NA | 100 |
| Rotermann (2017) | cardiac surgery | Germany | 62.13 | 39.20 | 57.18 | 56.94 | 0.24 | NA | NA | NA | NA | 2007 | 72.63 | 346.3 | NA | 72.63 | NA |
| Ryerson (2015) | ECMO | Canada | 55.1 | NA | 52.9 | 43.4 | 9.5 | NA | NA | NA | 100 | 2004 | NA | NA | NA | NA | 65 |
| Sarajuuri (2007) | cardiac surgery | Finland | NA | NA | 70.10 | 69.16 | 0.94 | NA | NA | NA | NA | NA | 100 | 121 | 100 | 100 | NA |
| Sarajuuri (2012) | cardiac surgery | Finland | NA | NA | 61.46 | 61.16 | 0.30 | NA | NA | NA | NA | 2003 | 100 | 174.1 | NA | 100 | NA |
| Sarrechia article 1 (2015) | cardiac surgery | Belgium | 41.30 | 40 | 106.96 | 90.78 | 16.17 | 2.39 | NA | NA | NA | 2004 | 100 | NA | NA | 0 | NA |
| Sarrechia article 2 (2015) | cardiac surgery | Belgium | 33.33 | 40 | 110 | 77 | 33 | NA | NA | NA | NA | NA | 100 | 39.9 | NA | 0 | NA |
| Schaefer (2013) | cardiac surgery | Switzerland | 42.37 | NA | 164.98 | 154.18 | 10.8 | NA | NA | NA | NA | 1996 | 100 | 85.9 | NA | 51 | NA |
| Schiller (2016) | ECMO | The Netherlands | 54.13 | 40.09 | 96 | 95.50 | 0.50 | NA | 17.22 | NA | 100 | 2001 | NA | NA | NA | NA | 73 |
| Shida (1981) | cardiac surgery | Japan | NA | NA | NA | NA | NA | NA | NA | NA | NA | NA | 100 | NA | 100 | 0 | 93 |
| Simons (2010) | cardiac surgery | USA | 61.29 | 38.1 | 72.35 | 62.25 | 10.1 | 2.4 | 0.02 | NA | NA | 2001 | 100 | 58.5 | 91 | 0 | NA |
| Singer (1989) | miscellaneous | USA | NA | 35.7 | 66 | 62.7 | 3.3 | NA | NA | NA | NA | 1977 | NA | NA | NA | NA | 71 |
| Slomine (2018) | CPR | USA | 60 | NA | 171.6 | 12 | 159.6 | 35.39 | NA | 100 | NA | 2012 | NA | NA | NA | NA | NA |
| Sorensen (2014) | miscellaneous | USA | 47.31 | NA | 102 | 84.33 | 17.67 | NA | NA | NA | NA | NA | NA | NA | NA | NA | NA |
| Stein (2013) | heart- or heart-lung tx | USA | 70 | NA | 166.80 | 65.07 | 101.73 | NA | NA | 19.80 | 9.90 | 1997 | 100 | NA | 100 | 15.0 | NA |
| Sugimoto (2013) | cardiac surgery | Japan | NA | NA | 105.24 | 79.22 | 26.02 | NA | NA | 12.6 | NA | 2005 | 100 | 70.4 | 69 | 100 | 93 |
| Urschel (2018) | heart- or heart-lung tx | Canada | 58.18 | NA | 54.58 | 38.89 | 15.69 | 32.71 | 17.51 | 25.46 | 22 | 2005 | 100 | NA | 100 | NA | 81 |
| Uzark (1998) | cardiac surgery | USA | 46.88 | NA | 75.6 | 37.24 | 38.36 | NA | NA | NA | NA | 1990 | 100 | 129 | 15.63 | 100 | 92 |
| Uzark (2009) | heart- or heart-lung tx | USA | 42.86 | NA | 40.72 | 14.35 | 25.4 | NA | NA | NA | NA | 2002 | 100 | NA | 100 | NA | NA |
| van der Rijken (2008) | cardiac surgery | The Netherlands | 46.51 | NA | 151.2 | 12 | 139.2 | NA | NA | NA | NA | 2004 | 100 | 207.7 | 0 | 34.88 | 98 |
| Venchiarutti (2019) | cardiac surgery | Italy | 52.94 | 38.6 | 117.12 | NA | NA | NA | NA | NA | NA | NA | 100 | NA | NA | 47.06 | NA |
| Vergine (2021) | cardiac surgery | Italy | 57.9 | 37.9 | 78 | 68.4 | 9.6 | 7 | NA | NA | NA | 2013 | 100 | 123 | NA | 68.4 | NA |
| Vermunt (2009) | sepsis and/or meningoencephalitis | The Netherlands | 52 | NA | 132 | 96 | 36 | 5.7 | NA | NA | NA | 1994 | NA | NA | NA | NA | NA |
| Vermunt (2011) | sepsis and/or meningoencephalitis | The Netherlands | 48 | NA | 256.8 | 149.76 | 107.04 | NA | NA | NA | NA | 1994 | NA | NA | NA | NA | NA |
| Volpe (2017) | TBI | Brazil | 56 | NA | 136.13 | 66.28 | 69.85 | 5.20 | 3.24 | NA | NA | 2009 | NA | NA | NA | NA | 91 |
| von Rhein (2012) | cardiac surgery | Switzerland | 56 | NA | 124.8 | 107.66 | 17.14 | 10.25 | NA | 8 | NA | 1996 | 100 | 94.5 | 9 | 43 | NA |
| Wells (1983) | cardiac surgery | UK | NA | NA | 68.73 | 53.37 | 15.37 | NA | NA | NA | NA | 1974 | 100 | NA | 63.27 | 57.2 | NA |
| Wernovsky (2000) | cardiac surgery | USA | 54.90 | NA | 169.20 | 81.60 | 87.60 | NA | NA | NA | NA | 1982 | 100 | 119.9 | NA | 100 | NA |
| Whitman (1973) | cardiac surgery | USA | NA | NA | 103.33 | 1.74 | 101.59 | NA | NA | NA | NA | 1972 | 61.11 | NA | NA | 16.67 | NA |
| Wolfe article 1 (2020) | heart- or heart-lung tx | USA | 51.85 | NA | 111.6 | 73.92 | 37.68 | NA | NA | NA | 22 | 2014 | 100 | NA | 100 | NA | NA |
| Wolfe article 2 (2020) | cardiac surgery | USA | 50.0 | NA | 114.6 | NA | NA | NA | NA | NA | NA | NA | 100 | NA | NA | 100 | NA |
| Wotherspoon (2020) | cardiac surgery | Australia | 47.6 | 39.22 | 184.8 | 183.26 | 1.54 | NA | NA | NA | NA | 2000 | 100 | NA | 14.3 | 71.43 | 100 |
| Wray (1994) | cardiac surgery | UK and Ireland | NA | NA | 74.4 | NA | NA | NA | NA | NA | NA | NA | NA | NA | NA | NA | NA |
| Wray (1994) | heart- or heart-lung tx | UK and Ireland | NA | NA | 112.8 | 10 | 102.8 | NA | NA | NA | NA | 1989 | 100 | NA | 100 | NA | NA |
| Wray article 1 (2001) | heart- or heart-lung tx | UK and Ireland | 48 | NA | 154.44 | 36 | 118.44 | NA | NA | NA | NA | 1990 | 100 | NA | 100 | NA | 77 |
| Wray article 2 (2001) | cardiac surgery | UK | 42.55 | NA | 102.3 | 13.5 | 88.8 | NA | NA | NA | NA | NA | 85 | NA | NA | 36.2 | 90 |
| Wray (2005) | heart- or heart-lung tx | UK and Ireland | 51.06 | NA | 111.6 | 12 | 99.6 | NA | NA | NA | NA | NA | 100 | NA | 100 | NA | NA |
| Wray (2006) | heart- or heart-lung tx | UK | 52.94 | NA | NA | 36 | NA | NA | NA | NA | NA | NA | 100 | NA | 100 | NA | NA |
| Wright (1994) | cardiac surgery | Australia | 68.97 | 39.3 | 114 | 103.37 | 10.63 | NA | NA | NA | NA | 1981 | 100 | 16.7 | 100 | 100 | NA |
| Note: If a study included different subgroups based on reasons of PICU admission, subgroups are displayed in different rows. CPR = cardiopulmonary resuscitation; CPB = cardiopulmonary bypass (during cardiac surgery); DHCA = deep-hypothermic circulatory arrest (during cardiac surgery); ECMO = extra-corporeal membrane oxygenation, heart- or heart-lung tx = heart- or heart-lung transplantation; NA = not available; TBI = traumatic brain injury; UK = United Kingdom; USA = United States of America. Meta-regression was not possible to perform for Pediatric Risk of Mortality (PRISM) score, Pediatric Index of Mortality (PIM) 2 score and Vasopressor score because these variables were reported in less than ten studies (data not shown). | | | | | | | | | | | | | | | | | |

| **Table S2.** Study characteristics and FSIQ differences (Cohen’s *d*) between PICU survivors and controls. | | | | | | | |
| --- | --- | --- | --- | --- | --- | --- | --- |
| **Study name** | **Subgroup** | **Comparison** | **Cohen’s *d*** | **SE** | **Sample size PICU** | **Sample size Control** | **Study quality** |
| Als (2013) | sepsis and/or meningoencephalitis | control | -0.79 | 0.25 | 41 | 100 | 5 |
| Als (2013) | miscellaneous | control | -0.53 | 0.18 | 44 | 100 | 5 |
| Anderson (2021) | heart- or heart-lung tx | normative | -1.44 | 0.40 | 54 | 54 | 5 |
| Asschenfeldt (2020) | cardiac surgery | control | -0.87 | 0.21 | 66 | 40 | 5 |
| Atallah (2020) | cardiac surgery | normative | -0.86 | 0.18 | 68 | 68 | 5 |
| Baum (2000) | heart- or heart-lung tx | normative | -1.21 | 0.23 | 46 | 46 | 5 |
| Baum (2004) | heart- or heart-lung tx | normative | -1.11 | 0.20 | 55 | 55 | 4 |
| Bellinger (2003) | cardiac surgery | normative | -0.29 | 0.27 | 155 | 155 | 5 |
| Benjamin (2013) | miscellaneous | normative | -0.66 | 0.36 | 16 | 16 | 4 |
| Bergemann (2015) | cardiac surgery | control | -1.34 | 0.41 | 38 | 34 | 7 |
| Bouman (2000) | miscellaneous | normative | -1.00 | 0.47 | 10 | 10 | 4 |
| Brosig (2013) | cardiac surgery | normative | 0.01 | 0.24 | 34 | 34 | 4 |
| Cainelli (2021) | cardiac surgery | control | -0.57 | 0.25 | 35 | 33 | 5 |
| Calderon (2010) | cardiac surgery | control | -0.51 | 0.31 | 21 | 21 | 6 |
| Calderon (2013) | cardiac surgery | normative | 0.92 | 0.31 | 45 | 45 | 5 |
| Campbell (2004) | TBI | normative | -0.51 | 0.29 | 25 | 25 | 4 |
| Carra (2021) | cardiac surgery | normative | -0.56 | 0.16 | 87 | 87 | 4 |
| Claessens (2018) | cardiac surgery | normative | -0.37 | 0.26 | 30 | 30 | 5 |
| Cottrell (2004) | cardiac surgery | normative | -0.52 | 0.12 | 156 | 156 | 4 |
| Creighton (2007) | cardiac surgery | normative | -0.39 | 0.43 | 53 | 53 | 5 |
| de Ferranti (2004) | cardiac surgery | normative | -0.19 | 0.11 | 154 | 154 | 5 |
| DeMaso (2017) | cardiac surgery | control | -1.03 | 0.15 | 91 | 111 | 4 |
| Deng (2021) | sepsis and/or meningoencephalitis | normative | -0.41 | 0.43 | 11 | 11 | 4 |
| Desai (1999) | ECMO | normative | -0.29 | 0.26 | 62 | 62 | 5 |
| Dickinson (1979) | cardiac surgery | normative | -0.05 | 0.23 | 38 | 38 | 4 |
| du Plessis (2002) | cardiac surgery | normative | -1.26 | 0.49 | 10 | 10 | 3 |
| Dunbar-Masterson (2001) | cardiac surgery | normative | -0.19 | 0.11 | 155 | 155 | 5 |
| Eder (2021) | miscellaneous | normative | -0.82 | 0.27 | 31 | 31 | 5 |
| Ehrler (2020) | cardiac surgery | normative | -0.30 | 0.14 | 107 | 107 | 4 |
| Eichler (2019) | cardiac surgery | control | -0.59 | 0.23 | 39 | 39 | 6 |
| Fiser (2000) | miscellaneous | normative | -1.21 | 0.18 | 75 | 75 | 4 |
| Fleisher (2002) | cardiac surgery | normative | 0.05 | 0.45 | 10 | 10 | 4 |
| Fleisher (2002) | heart- or heart-lung tx | normative | -0.19 | 0.43 | 11 | 11 | 4 |
| Forbess (2002) | cardiac surgery | normative | -0.21 | 0.09 | 243 | 243 | 4 |
| Fourdain (2020) | cardiac surgery | normative | 0.03 | 0.22 | 80 | 80 | 4 |
| Glass (1997) | ECMO | control | -1.04 | 0.28 | 151 | 53 | 6 |
| Goff (2012) | cardiac surgery | normative | -0.29 | 0.07 | 378 | 378 | 4 |
| Gold article 1 (2020) | miscellaneous | normative | -0.30 | 0.47 | 21 | 21 | 5 |
| Gold article 2 (2020) | heart- or heart-lung tx | normative | -1.11 | 0.30 | 25 | 25 | 5 |
| Goldberg (2000) | cardiac surgery | normative | 0.10 | 0.20 | 48 | 48 | 4 |
| Guan (2011) | cardiac surgery | control | -0.38 | 0.36 | 16 | 16 | 6 |
| Guerra (2014) | cardiac surgery | normative | -0.49 | 0.15 | 91 | 91 | 5 |
| Guerra (2015) | CPR | normative | -1.52 | 0.39 | 17 | 17 | 4 |
| Haneda (1996) | cardiac surgery | normative | 0.41 | 0.49 | 82 | 82 | 4 |
| Hansen (2016) | cardiac surgery | normative | -0.46 | 0.22 | 42 | 42 | 5 |
| Heinrichs (2014) | cardiac surgery | normative | 0.34 | 0.19 | 56 | 56 | 4 |
| Heye (2019) | cardiac surgery | normative | -0.33 | 0.12 | 143 | 143 | 5 |
| Hiraiwa article 1 (2020) | cardiac surgery | normative | -0.50 | 0.24 | 35 | 35 | 5 |
| Hiraiwa article 2 (2020) | cardiac surgery | control | -0.82 | 0.34 | 27 | 39 | 5 |
| Hofkosh (1991) | ECMO | Combined | -0.26 | 0.47 | 19 | 19 | 4 |
| Hövels-Gürich (2002) | cardiac surgery | normative | -0.07 | 0.18 | 60 | 60 | 4 |
| Ikle (2003) | heart- or heart-lung tx | normative | -0.82 | 0.41 | 13 | 13 | 4 |
| Iwamoto (1990) | cardiac surgery | normative | -0.21 | 0.49 | 75 | 75 | 4 |
| Jacobs (2020) | miscellaneous | control | -0.76 | 0.07 | 684 | 369 | 6 |
| Jin (2018) | cardiac surgery | control | -0.38 | 0.34 | 18 | 18 | 6 |
| Jones (2015) | cardiac surgery | normative | -0.29 | 0.32 | 20 | 20 | 4 |
| Karl (2004) | cardiac surgery | control | -0.54 | 0.17 | 74 | 74 | 6 |
| Kaur (2016) | sepsis and/or meningoencephalitis | control | -0.52 | 0.24 | 35 | 35 | 6 |
| Kern (1998) | cardiac surgery | normative | -1.30 | 0.45 | 12 | 12 | 5 |
| King (2017) | cardiac surgery | control | -0.57 | 0.35 | 17 | 17 | 5 |
| Kirshbom (2005) | cardiac surgery | normative | -0.28 | 0.26 | 30 | 30 | 4 |
| Krueger (2015) | cardiac surgery | normative | -0.50 | 0.12 | 146 | 146 | 5 |
| Krull (2003) | miscellaneous | normative | -0.57 | 0.37 | 15 | 15 | 4 |
| Langenbacher (2001) | ECMO | normative | -0.45 | 0.20 | 49 | 49 | 4 |
| Latal article 1 (2016) | cardiac surgery | normative | -0.19 | 0.20 | 48 | 48 | 5 |
| Latal article 2 (2016) | cardiac surgery | control | -0.67 | 0.23 | 48 | 32 | 6 |
| Leeuwen (2018) | ECMO | normative | -0.82 | 0.40 | 35 | 35 | 5 |
| Leeuwen (2018) | miscellaneous | normative | 0.00 | 0.26 | 30 | 30 | 5 |
| Ma (2020) | cardiac surgery | control | -1.53 | 0.48 | 10 | 13 | 5 |
| Madderom (2013) | ECMO | normative | -0.48 | 0.38 | 14 | 14 | 5 |
| Madderom (2013) | miscellaneous | normative | 0.64 | 0.37 | 15 | 15 | 5 |
| Madderom (2016) | ECMO | normative | -0.56 | 0.28 | 27 | 27 | 4 |
| Mahle (2006) | cardiac surgery | normative | -1.01 | 0.31 | 47 | 47 | 4 |
| Majnemer (2008) | cardiac surgery | normative | -0.50 | 0.15 | 94 | 94 | 5 |
| Melchers (1999) | TBI | normative | -0.74 | 0.52 | 19 | 19 | 4 |
| Mesotten (2012) | miscellaneous | control | -0.84 | 0.10 | 456 | 216 | 5 |
| Miatton (2008) | cardiac surgery | control | -0.75 | 0.22 | 43 | 43 | 7 |
| Mittnacht (2015) | cardiac surgery | normative | -0.08 | 0.27 | 28 | 28 | 5 |
| Morris (1993) | CPR | normative | -0.80 | 0.29 | 25 | 25 | 4 |
| Muñoz-López (2017) | cardiac surgery | normative | -0.21 | 0.22 | 40 | 40 | 4 |
| Murphy (2017) | cardiac surgery | control | -0.61 | 0.34 | 18 | 18 | 6 |
| Naef (2017) | cardiac surgery | normative | -0.34 | 0.11 | 169 | 169 | 5 |
| Naguib (2015) | cardiac surgery | normative | -0.76 | 0.60 | 20 | 20 | 4 |
| Neufeld (2008) | cardiac surgery | normative | -0.31 | 0.18 | 65 | 65 | 4 |
| Nijhuis-van der Sanden (2009) | ECMO | normative | -0.02 | 0.12 | 131 | 131 | 5 |
| Oates (1995) | cardiac surgery | normative | 0.05 | 0.23 | 168 | 168 | 5 |
| Oberhuber (2017) | cardiac surgery | normative | -0.85 | 0.23 | 43 | 43 | 4 |
| Omeje (2003) | cardiac surgery | normative | -0.05 | 0.18 | 64 | 64 | 4 |
| Poncelet (2011) | cardiac surgery | normative | 0.19 | 0.48 | 18 | 18 | 4 |
| Quartermain (2010) | cardiac surgery | control | 0.56 | 0.34 | 32 | 12 | 5 |
| Quartermain (2010) | miscellaneous | control | 0.59 | 0.38 | 18 | 12 | 5 |
| Rotermann (2017) | cardiac surgery | normative | -0.61 | 0.24 | 95 | 95 | 5 |
| Ryerson (2015) | ECMO | normative | -1.28 | 0.23 | 44 | 44 | 5 |
| Sarajuuri (2007) | cardiac surgery | normative | -0.82 | 0.47 | 26 | 26 | 5 |
| Sarajuuri (2012) | cardiac surgery | control | -1.41 | 0.32 | 36 | 40 | 6 |
| Sarrechia article 1 (2015) | cardiac surgery | control | -0.57 | 0.32 | 46 | 46 | 6 |
| Sarrechia article 2 (2015) | cardiac surgery | control | -0.89 | 0.29 | 18 | 48 | 6 |
| Schaefer (2013) | cardiac surgery | control | -0.67 | 0.21 | 59 | 40 | 6 |
| Schiller (2016) | ECMO | normative | -0.13 | 0.20 | 178 | 178 | 5 |
| Shida (1981) | cardiac surgery | normative | 0.69 | 0.22 | 45 | 45 | 4 |
| Simons (2010) | cardiac surgery | normative | -0.29 | 0.26 | 31 | 31 | 4 |
| Singer (1989) | miscellaneous | normative | -0.54 | 0.25 | 32 | 32 | 5 |
| Slomine (2018) | CPR | normative | -0.55 | 0.23 | 41 | 41 | 5 |
| Sorensen (2014) | miscellaneous | normative | -0.53 | 0.15 | 91 | 91 | 4 |
| Stein (2013) | heart- or heart-lung tx | control | -1.01 | 0.46 | 20 | 12 | 6 |
| Sugimoto (2013) | cardiac surgery | normative | -1.01 | 0.18 | 70 | 70 | 4 |
| Urschel (2018) | heart- or heart-lung tx | normative | -1.31 | 0.30 | 55 | 55 | 5 |
| Uzark (1998) | cardiac surgery | normative | -0.18 | 0.25 | 32 | 32 | 4 |
| Uzark (2009) | heart- or heart-lung tx | normative | -0.89 | 0.32 | 21 | 21 | 5 |
| van der Rijken (2008) | cardiac surgery | control | -0.28 | 0.22 | 43 | 43 | 6 |
| Venchiarutti (2019) | cardiac surgery | normative | -0.06 | 0.34 | 17 | 17 | 4 |
| Vergine (2021) | cardiac surgery | normative | -0.02 | 0.23 | 38 | 38 | 4 |
| Vermunt (2009) | sepsis and/or meningoencephalitis | normative | -0.20 | 0.17 | 66 | 66 | 4 |
| Vermunt (2011) | sepsis and/or meningoencephalitis | normative | -0.26 | 0.21 | 46 | 46 | 4 |
| Volpe (2017) | TBI | normative | -1.56 | 0.48 | 25 | 25 | 4 |
| von Rhein (2012) | cardiac surgery | normative | -0.68 | 0.13 | 117 | 117 | 5 |
| Wells (1983) | cardiac surgery | control | -1.31 | 0.44 | 49 | 29 | 5 |
| Wernovsky (2000) | cardiac surgery | normative | -0.35 | 0.26 | 93 | 93 | 4 |
| Whitman (1973) | cardiac surgery | normative | 0.55 | 0.49 | 18 | 18 | 4 |
| Wolfe article 1 (2020) | heart- or heart-lung tx | normative | -1.20 | 0.30 | 27 | 27 | 4 |
| Wolfe article 2 (2020) | cardiac surgery | normative | -0.67 | 0.18 | 66 | 66 | 4 |
| Wotherspoon (2020) | cardiac surgery | normative | -0.41 | 0.32 | 20 | 20 | 4 |
| Wray (1994) | cardiac surgery | control | -0.08 | 0.24 | 35 | 35 | 5 |
| Wray (1994) | heart- or heart-lung tx | control | -0.83 | 0.23 | 49 | 35 | 5 |
| Wray article 1 (2001) | heart- or heart-lung tx | normative | 0.14 | 0.28 | 25 | 25 | 4 |
| Wray article 2 (2001) | cardiac surgery | control | -0.09 | 0.22 | 35 | 51 | 7 |
| Wray (2005) | heart- or heart-lung tx | control | -0.63 | 0.24 | 35 | 35 | 6 |
| Wray (2006) | heart- or heart-lung tx | normative | 0.01 | 0.30 | 22 | 22 | 4 |
| Wright (1994) | cardiac surgery | control | -0.56 | 0.25 | 29 | 36 | 6 |
| Note: If a study included different subgroups based on reasons of PICU admission, subgroups are displayed in different rows. CPR = cardiopulmonary resuscitation; ECMO = extra-corporeal membrane oxygenation, heart- or heart-lung tx = heart- or heart-lung transplantation; TBI = traumatic brain injury; control = healthy control group; normative = normative data (i.e. mean = 100, SD = 15); Study quality assessed by the Newcastle Ottawa Scale for cohort studies, revised to a maximum of 7 points. | | | | | | | |

| **Table S3.** Results of univariate meta-regression analyses of risk factors for FSIQ impairment in PICU subgroups. | | | | | |
| --- | --- | --- | --- | --- | --- |
| **Risk factors** | ***k*** | **Beta** | **95% CI** | **R**^2^ **(%)** | **Range studied** |
| **Respiratory and/or circulatory insufficiency necessitating ECMO** |  |  |  |  |  |
| Year of PICU admission | 10 | -0.012 | -0.055, 0.031 | 0 | 1983-2007 |
| Age at follow-up (months) | 10 | 0.002 | -0.010, 0.014 | 0 | 52.9-144.1 |
| Study quality | 10 | -0.209 | -0.734, 0.317 | 0 | 4-6 |
| **Cardiac surgery** |  |  |  |  |  |
| Year of PICU admission | 64 | -0.012 ** | -0.020, -0.003 | 12 | 1972-2013 |
| Sex (% boys) | 65 | 0.008 * | 0.000, 0.015 | 6 | 30.3-79.4 |
| Gestational age (weeks) | 34 | -0.134 | -0.309, 0.042 | 0 | 37.9-40.0 |
| Age at PICU admission (months) | 67 | 0.002 | -0.001, 0.004 | 0 | 0.2-141.6 |
| PICU stay (days) | 22 | -0.011 | -0.031, 0.009 | 0 | 1.3-25.3 |
| Resuscitation (%) | 13 | 0.001 | -0.051, 0.052 | 0 | 0.0-14.6 |
| Duration of CPB during surgery   (minutes) | 49 | 0.000 | -0.001, 0.002 | 0 | 17-385 |
| DHCA (%) | 41 | 0.001 | -0.002, 0.004 | 0 | 0.0-100 |
| Cyanotic heart disease (%) | 71 | -0.002 | -0.005, 0.001 | 0 | 0.0-100 |
| Rate of survivors (%) | 34 | 0.010 | -0.000, 0.020 | 9 | 57.0-100 |
| Age at follow-up (months) | 75 | -0.001 | -0.003, 0.001 | 0 | 30.1-307.2 |
| Time to follow-up (months) | 69 | -0.002 | -0.004, 0.000 | 0 | 0.7-231.6 |
| Study quality | 80 | -0.148 ** | -0.243, -0.053 | 9 | 3-7 |
| **Heart- or heart-lung transplantation** |  |  |  |  |  |
| Year of PICU admission | 10 | -0.035 ** | -0.061, -0.009 | 65 | 1989-2016 |
| Sex (% boys) | 12 | -0.001 | -0.027, 0.025 | 0 | 28.0-80.8 |
| Age at PICU admission (months) | 11 | 0.006 ** | 0.002, 0.011 | 74 | 1.6-118.4 |
| Age at follow-up (months) | 11 | 0.008 ** | 0.002, 0.015 | 68 | 40.7-166.8 |
| Time to follow-up (months) | 12 | -0.006 | -0.016, 0.004 | 8 | 10.0-82.3 |
| Study quality | 14 | -0.154 | -0.537, 0.228 | 0 | 4-6 |
| Note: CPB = cardiopulmonary bypass; DHCA = deep-hypothermic circulatory arrest (during cardiac surgery); ECMO = extra-corporeal membrane oxygenation; PICU = pediatric intensive care unit. Study quality assessed by the Newcastle Ottawa Scale for cohort studies, revised to a maximum of 7 points. Unstandardized Beta’s are reported. There was limited variation in percentage use of cardiopulmonary bypass since almost all articles focusing on cardiac surgery reported 100% use of cardiopulmonary bypass and all articles focusing on heart- or heart-lung transplantation reported 100% use of cardiopulmonary bypass. Therefore, this variable was omitted from meta-regression. Furthermore, there was limited variation in percentage use of deep hypothermic circulatory arrest in articles focusing on heart- or heart-lung transplantation and therefore this variable was omitted from meta-regression in this subgroup. *p<.05. **p<.01. ***p<.001. | | | | | |

**Study quality**

Study quality was assessed using the Newcastle-Ottawa Scale for cohort studies (24), that assesses aspects of participant selection, group comparability and outcome assessment. Two items of the scale (“Demonstration that outcome of interest was not present at the start of study” and “Was follow-up long enough for outcomes to occur?”) were not applicable and therefore omitted. Furthermore, the scoring of some items was adjusted to fit the aim of the current meta-analysis. “Selection of the non-exposed cohort” was awarded one point if a healthy control group was included. Comparability was awarded one point if the healthy control group was matched on socioeconomic status since this is known to be an important factor in intelligence (25, 26), and one point was assigned for comparability if the control group was matched on age and/or sex. If FSIQ was measured in at least 70% of the total sample, one point was awarded for “adequacy of follow up of cohorts”. All included studies were independently rated by two authors. Any disagreements were solved through discussion or by consulting a third author.

**Search strategy**

("Extracorporeal Membrane Oxygenation"[Mesh] OR extracorporeal membrane oxygenation*[tiab] OR ECMO*[tiab] OR extracorporeal life support*[tiab] OR ECLS*[tiab] OR extra corporeal membrane oxygenation*[tiab] OR extracorporeal pump oxygenation*[tiab] OR extrapulmonary oxygenation*[tiab] OR extracorporeal oxygenation*[tiab] OR "Thoracic Surgical Procedures"[Mesh] OR "Thoracic Surgery"[Mesh] OR "Thorax/surgery"[Mesh] OR cardiac surg*[tiab] OR heart surg*[tiab] OR thoracic surg*[tiab] OR cardiac surg*[tiab] OR heart surg*[tiab] OR cardiothoracic surg*[tiab] OR chest surg*[tiab] OR chest wall surg*[tiab] OR thorax surg*[tiab] OR thoracic operation*[tiab] OR "Critical Care"[Mesh] OR "Intensive Care Units, Pediatric"[Mesh] OR "Critical Illness"[Mesh] OR intensive care[tiab] OR PICU*[tiab] OR IC[tiab] OR ICU*[tiab] OR critical ill*[tiab] OR critically ill*[tiab])

AND

("Child"[Mesh] OR "Adolescent"[Mesh] OR "Infant"[Mesh:NoExp] OR "Infant, Newborn"[Mesh:NoExp] OR "Pediatrics"[Mesh] OR child*[tiab] OR pediatric*[tiab] OR paediatric*[tiab] OR infant*[tiab] OR adoles*[tiab] OR teen*[tiab] OR youth*[tiab] OR schoolchild*[tiab] OR preschool[tiab] OR pre-school[tiab] OR kid[tiab] OR kids[tiab] OR toddler*[tiab] OR juvenil*[tiab] OR teen*[tiab] OR pubescen*[tiab] OR puber*[tiab] OR prepubert*[tiab] OR school age*[tiab] OR schoolage*[tiab] OR boy*[tiab] OR girl*[tiab] OR underag*[tiab] OR under ag*[tiab])

AND

("Neurocognitive Disorders"[Mesh] OR "Cognition"[Mesh] OR "Neuropsychology"[Mesh] OR "Intelligence"[Mesh] OR neurocogniti*[tiab] OR cogniti*[tiab] OR neurodevelopment*[tiab] OR neuropsycholog*[tiab] OR intelligence[tiab] OR intellectual[tiab] OR IQ[tiab])

NOT

("fetal growth retardation"[Mesh] OR fetal growth retardat*[tiab] OR fetal growth restrict*[tiab] OR intrauterine growth restrict*[tiab] OR small for gestation*[tiab] OR Intrauterine Growth retardat*[tiab] OR prematur*[tiab] OR preterm[tiab] OR low birth weigh*[tiab] OR SGA[tiab] OR IUGR[tiab])
