# Supplementary figures and images for: Intelligence outcome of pediatric intensive care unit survivors: a systematic meta-analysis and meta-regression
Source: BMC Med. 2022 Jun 1;20:198. doi: 10.1186/s12916-022-02390-5 (PMC9158152; doi:10.1186/s12916-022-02390-5)

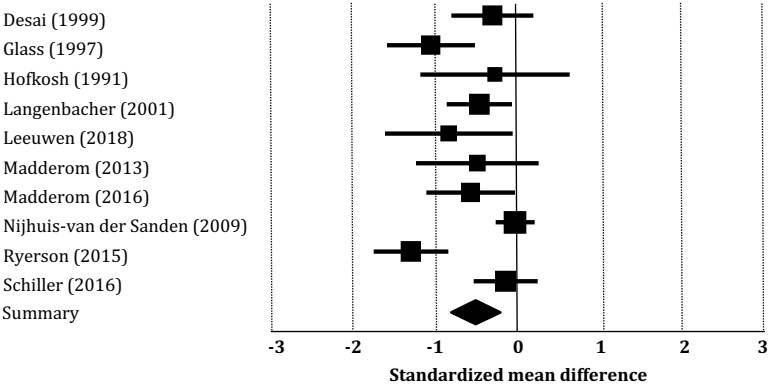

Supplement: Supplementary file 2 — Additional file 2. Figure S1 - Forest plot showing standardized mean differences and accompanying 95% CI of studies reporting on the subgroup Respiratory and/or circulatory insufficiency necessitating ECMO, comparing FSIQ of PICU survivors to healthy controls or normative data. [file 12916_2022_2390_MOESM2_ESM.pdf]

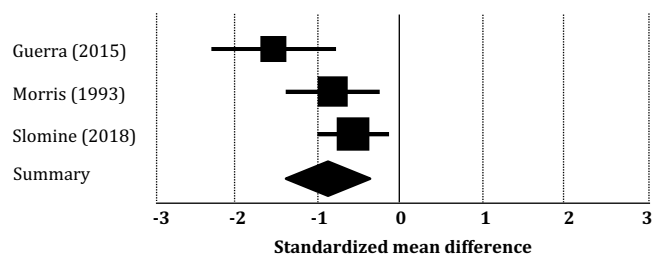

Supplement: Supplementary file 3 — Additional file 3. Figure S2 - Forest plot showing standardized mean differences and accompanying 95% CI of studies reporting on the subgroup Circulatory insufficiency necessitating CPR, comparing FSIQ of PICU survivors to healthy controls or normative data. [file 12916_2022_2390_MOESM3_ESM.pdf]

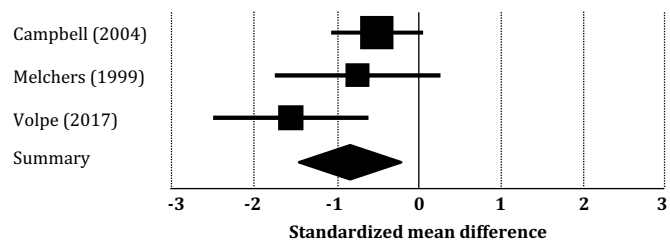

Supplement: Supplementary file 4 — Additional file 4. Figure S3 - Forest plot showing standardized mean differences and accompanying 95% CI of studies reporting on the subgroup Traumatic brain injury, comparing FSIQ of PICU survivors to healthy controls or normative data. [file 12916_2022_2390_MOESM4_ESM.pdf]

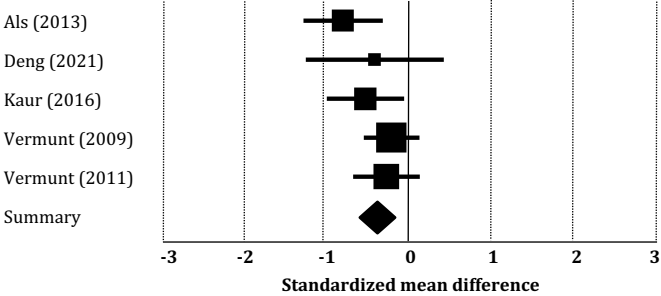

Supplement: Supplementary file 5 — Additional file 5. Figure S4 - Forest plot showing standardized mean differences and accompanying 95% CI of studies reporting on the subgroup Sepsis and/or meningoencephalitis, comparing FSIQ of PICU survivors to healthy controls or normative data. [file 12916_2022_2390_MOESM5_ESM.pdf]

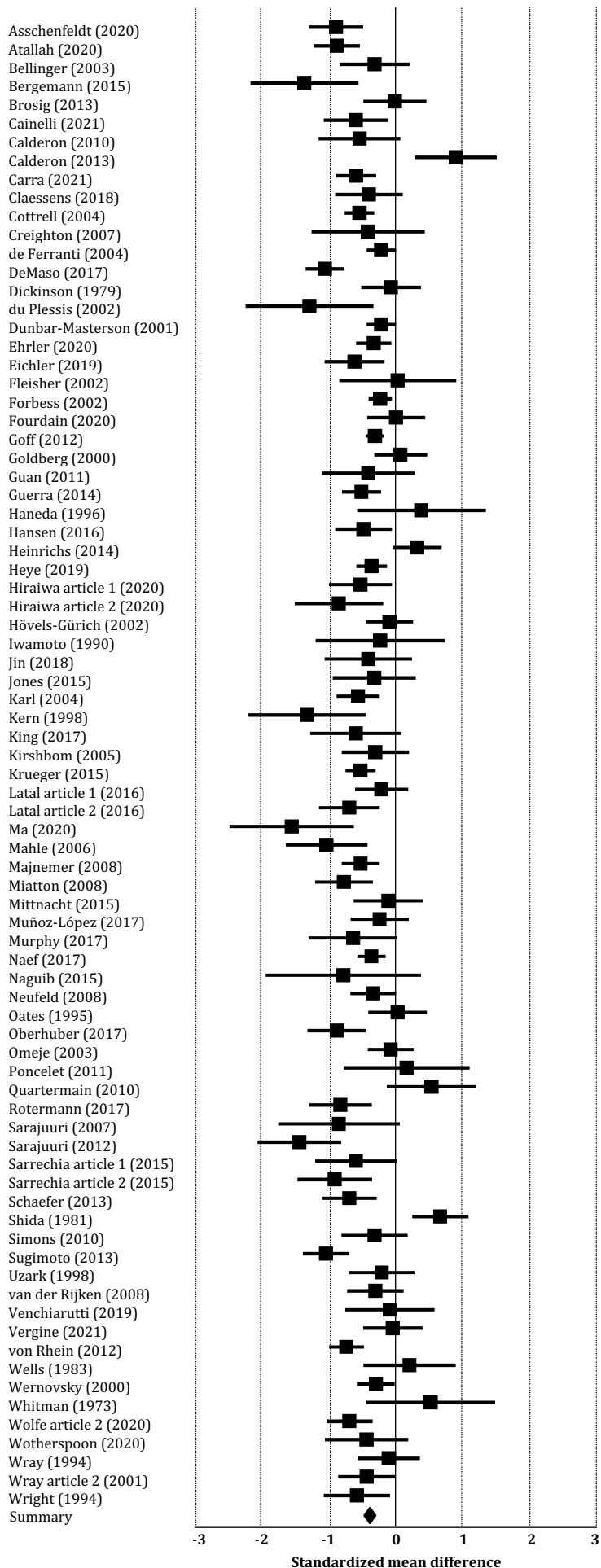

Supplement: Supplementary file 6 — Additional file 6. Figure S5 - Forest plot showing standardized mean differences and accompanying 95% CI of studies reporting on the subgroup Cardiac surgery, comparing FSIQ of PICU survivors to healthy controls or normative data. [file 12916_2022_2390_MOESM6_ESM.pdf]

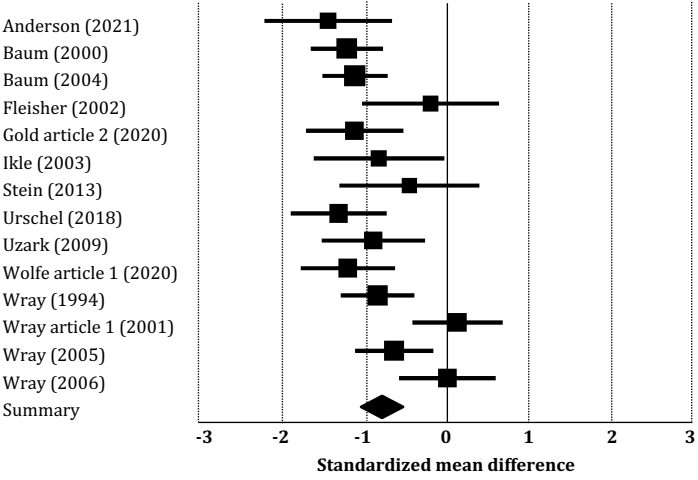

Supplement: Supplementary file 7 — Additional file 7. Figure S6 - Forest plot showing standardized mean differences and accompanying 95% CI of studies reporting on the subgroup Heart- or heart-lung transplantation, comparing FSIQ of PICU survivors to healthy controls or normative data. [file 12916_2022_2390_MOESM7_ESM.pdf]

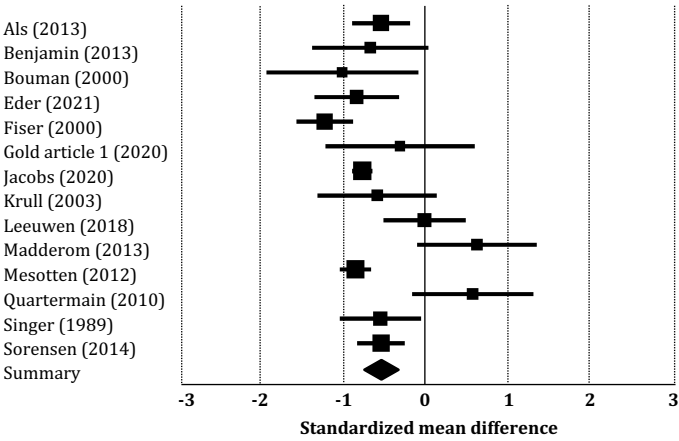

Supplement: Supplementary file 8 — Additional file 8. Figure S7 - Forest plot showing standardized mean differences and accompanying 95% CI of studies reporting on the subgroup Miscellaneous PICU admission indications, comparing FSIQ of PICU survivors to healthy controls or normative data. [file 12916_2022_2390_MOESM8_ESM.pdf]

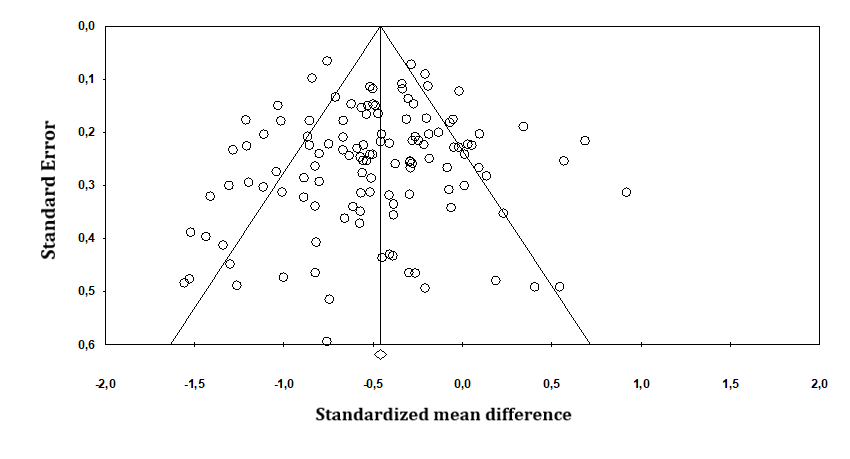

Supplement: Supplementary file 9 — Additional file 9. Figure S8 - Funnel plot of the study’s individual effect sizes for FSIQ plotted against its standard error. [file 12916_2022_2390_MOESM9_ESM.tif]
