# Supplementary material for: Intelligence outcome of pediatric intensive care unit survivors: a systematic meta-analysis and meta-regression
Source: BMC Med. 2022 Jun 1;20:198. doi: 10.1186/s12916-022-02390-5 (PMC9158152; doi:10.1186/s12916-022-02390-5)
Supplement: Supplementary file 10 — Additional file 10. References of the studies included in the meta-analysis. [file 12916_2022_2390_MOESM10_ESM.docx]

**REFERENCES OF THE STUDIES INCLUDED IN THE META-ANALYSIS**

1. Als LC, Nadel S, Cooper M, Pierce CM, Sahakian BJ, Garralda ME. Neuropsychologic function three to six months following admission to the PICU with meningoencephalitis, sepsis, and other disorders: a prospective study of school-aged children. Crit Care Med. 2013;41(4):1094-103.

2. Anderson NM, Bond GY, Joffe AR, MacDonald C, Robertson C, Urschel S, et al. Post-operative fluid overload as a predictor of hospital and long-term outcomes in a pediatric heart transplant population. Pediatr Transplant. 2021;25(3):e13897.

3. Asschenfeldt B, Evald L, Heiberg J, Salvig C, Østergaard L, Dalby RB, et al. Neuropsychological Status and Structural Brain Imaging in Adults With Simple Congenital Heart Defects Closed in Childhood. J Am Heart Assoc. 2020;9(11):e015843.

4. Atallah J, Garcia Guerra G, Joffe AR, Bond GY, Islam S, Ricci MF, et al. Survival, Neurocognitive, and Functional Outcomes After Completion of Staged Surgical Palliation in a Cohort of Patients With Hypoplastic Left Heart Syndrome. J Am Heart Assoc. 2020;9(4):e013632.

5. Baum M, Freier MC, Freeman KR, Chinnock RE. Developmental outcomes and cognitive functioning in infant and child heart transplant recipients. Prog Pediatr Cardiol. 2000;11(2):159-63.

6. Baum M, Freier MC, Freeman K, Babikian T, Ashwal S, Chinnock R, et al. Neuropsychological outcome of infant heart transplant recipients. J Pediatr. 2004;145(3):365-72.

7. Bellinger DC, Wypij D, duPlessis AJ, Rappaport LA, Jonas RA, Wernovsky G, et al. Neurodevelopmental status at eight years in children with dextro-transposition of the great arteries: the Boston Circulatory Arrest Trial. J Thorac Cardiovasc Surg. 2003;126(5):1385-96.

8. Benjamin JR, Gustafson KE, Smith PB, Ellingsen KM, Tompkins KB, Goldberg RN, et al. Perinatal factors associated with poor neurocognitive outcome in early school age congenital diaphragmatic hernia survivors. J Pediatr Surg. 2013;48(4):730-7.

9. Bergemann A, Hansen JH, Rotermann I, Voges I, Scheewe J, Otto-Morris C, et al. Neuropsychological performance of school-aged children after staged surgical palliation of hypoplastic left heart syndrome. Eur J Cardiothorac Surg. 2015;47(5):803-11.

10. Bouman NH, Koot HM, Tibboel D, Hazebroek FW. Children with congenital diaphragmatic hernia are at risk for lower levels of cognitive functioning and increased emotional and behavioral problems. Eur J Pediatr Surg. 2000;10(1):3-7.

11. Brosig C, Mussatto K, Hoffman G, Hoffmann RG, Dasgupta M, Tweddell J, et al. Neurodevelopmental outcomes for children with hypoplastic left heart syndrome at the age of 5 years. Pediatr Cardiol. 2013;34(7):1597-604.

12. Cainelli E, Bisiacchi PS, Cogo P, Padalino M, Simonato M, Vergine M, et al. Detecting neurodevelopmental trajectories in congenital heart diseases with a machine-learning approach. Sci Rep. 2021;11(1):2574.

13. Calderon J, Bonnet D, Courtin C, Concordet S, Plumet MH, Angeard N. Executive function and theory of mind in school-aged children after neonatal corrective cardiac surgery for transposition of the great arteries. Dev Med Child Neurol. 2010;52(12):1139-44.

14. Calderon J, Bonnet D, Pinabiaux C, Jambaqué I, Angeard N. Use of early remedial services in children with transposition of the great arteries. J Pediatr. 2013;163(4):1105-10.e1.

15. Campbell CG, Kuehn SM, Richards PM, Ventureyra E, Hutchison JS. Medical and cognitive outcome in children with traumatic brain injury. Can J Neurol Sci. 2004;31(2):213-9.

16. Carra G, Flechet M, Jacobs A, Verstraete S, Vlasselaers D, Desmet L, et al. Postoperative Cerebral Oxygen Saturation in Children After Congenital Cardiac Surgery and Long-Term Total Intelligence Quotient: A Prospective Observational Study. Crit Care Med. 2021;49(6):967-76.

17. Claessens NHP, Algra SO, Ouwehand TL, Jansen NJG, Schappin R, Haas F, et al. Perioperative neonatal brain injury is associated with worse school-age neurodevelopment in children with critical congenital heart disease. Dev Med Child Neurol. 2018;60(10):1052-8.

18. Cottrell SM, Morris KP, Davies P, Bellinger DC, Jonas RA, Newburger JW. Early postoperative body temperature and developmental outcome after open heart surgery in infants. Ann Thorac Surg. 2004;77(1):66-71

19. Creighton DE, Robertson CM, Sauve RS, Moddemann DM, Alton GY, Nettel-Aguirre A, et al. Neurocognitive, functional, and health outcomes at 5 years of age for children after complex cardiac surgery at 6 weeks of age or younger. Pediatrics. 2007;120(3):e478-86.

20. de Ferranti S, Gauvreau K, Hickey PR, Jonas RA, Wypij D, du Plessis A, et al. Intraoperative hyperglycemia during infant cardiac surgery is not associated with adverse neurodevelopmental outcomes at 1, 4, and 8 years. Anesthesiology. 2004;100(6):1345-52.

21. DeMaso DR, Calderon J, Taylor GA, Holland JE, Stopp C, White MT, et al. Psychiatric Disorders in Adolescents With Single Ventricle Congenital Heart Disease. Pediatrics. 2017;139(3).

22. Deng L, Barton B, Lorenzo J, Rashid H, Dastouri F, Booy R. Longer term outcomes following serogroup B invasive meningococcal disease. J Paediatr Child Health. 2021;57(6):894-902.

23. Desai SA, Stanley C, Gringlas M, Merton DA, Wolfson PJ, Needleman L, et al. Five-year follow-up of neonates with reconstructed right common carotid arteries after extracorporeal membrane oxygenation. J Pediatr. 1999;134(4):428-33.

24. Dickinson DF, Sambrooks JE. Intellectual performance in children after circulatory arrest with profound hypothermia in infancy. Arch Dis Child. 1979;54(1):1-6.

25. du Plessis AJ, Bellinger DC, Gauvreau K, Plumb C, Newburger JW, Jonas RA, et al. Neurologic outcome of choreoathetoid encephalopathy after cardiac surgery. Pediatr Neurol. 2002;27(1):9-17.

26. Dunbar-Masterson C, Wypij D, Bellinger DC, Rappaport LA, Baker AL, Jonas RA, et al. General health status of children with D-transposition of the great arteries after the arterial switch operation. Circulation. 2001;104(12 Suppl 1):I138-42.

27. Eder B, Melter M, Gabler V, Zant R, Knoppke B. Risk factors associated with cognitive impairment in patients after pediatric liver transplantation. Pediatr Transplant. 2021;25(2):e13879.

28. Ehrler M, Latal B, Polentarutti S, von Rhein M, Held L, Wehrle FM. Pitfalls of using IQ short forms in neurodevelopmental disorders: a study in patients with congenital heart disease. Pediatr Res. 2020;87(5):917-23.

29. Eichler A, Köhler-Jonas N, Stonawski V, Purbojo A, Moll GH, Heinrich H, et al. Child neurodevelopment and mental health after surgical ventricular septal defect repair: risk and protective factors. Dev Med Child Neurol. 2019;61(2):152-60.

30. Fiser DH, Long N, Roberson PK, Hefley G, Zolten K, Brodie-Fowler M. Relationship of pediatric overall performance category and pediatric cerebral performance category scores at pediatric intensive care unit discharge with outcome measures collected at hospital discharge and 1- and 6-month follow-up assessments. Crit Care Med. 2000;28(7):2616-20.

31. Fleisher BE, Baum D, Brudos G, Burge M, Carson E, Constantinou J, et al. Infant heart transplantation at Stanford: growth and neurodevelopmental outcome. Pediatrics. 2002;109(1):1-7.

32. Forbess JM, Visconti KJ, Hancock-Friesen C, Howe RC, Bellinger DC, Jonas RA. Neurodevelopmental outcome after congenital heart surgery: results from an institutional registry. Circulation. 2002;106(12 Suppl 1):I95-102.

33. Fourdain S, Caron-Desrochers L, Simard MN, Provost S, Doussau A, Gagnon K, et al. Impacts of an Interdisciplinary Developmental Follow-Up Program on Neurodevelopment in Congenital Heart Disease: The CINC Study. Front Pediatr. 2020;8:539451.

34. Glass P, Bulas DI, Wagner AE, Rajasingham SR, Civitello LA, Papero PH, et al. Severity of brain injury following neonatal extracorporeal membrane oxygenation and outcome at age 5 years. Dev Med Child Neurol. 1997;39(7):441-8.

35. Goff DA, Luan X, Gerdes M, Bernbaum J, D'Agostino JA, Rychik J, et al. Younger gestational age is associated with worse neurodevelopmental outcomes after cardiac surgery in infancy. J Thorac Cardiovasc Surg. 2012;143(3):535-42.

36. Gold A, Young JM, Solomon M, Grasemann H. Neuropsychological outcomes following pediatric lung transplantation. Pediatr Pulmonol. 2020;55(9):2427-36.

37. Gold A, Bondi BC, Ashkanase J, Dipchand AI. Early school-age cognitive performance post-pediatric heart transplantation. Pediatr Transplant. 2020;24(8):e13832.

38. Goldberg CS, Schwartz EM, Brunberg JA, Mosca RS, Bove EL, Schork MA, et al. Neurodevelopmental outcome of patients after the fontan operation: A comparison between children with hypoplastic left heart syndrome and other functional single ventricle lesions. J Pediatr. 2000;137(5):646-52.

39. Guan GT, Jin YP, Zheng RP, Liu FQ, Wang YL. Cognitive P300-evoked potentials in school-age children after surgical or transcatheter intervention for ventricular septal defect. Pediatr Int. 2011;53(6):995-1001.

40. Garcia Guerra G, Robertson CM, Alton GY, Joffe AR, Cave DA, Yasmin F, et al. Neurotoxicity of sedative and analgesia drugs in young infants with congenital heart disease: 4-year follow-up. Paediatr Anaesth. 2014;24(3):257-65.

41. Garcia Guerra G, Zorzela L, Robertson CM, Alton GY, Joffe AR, Moez EK, et al. Survival and neurocognitive outcomes in pediatric extracorporeal-cardiopulmonary resuscitation. Resuscitation. 2015;96:208-13.

42. Haneda K, Itoh T, Togo T, Ohmi M, Mohri H. Effects of cardiac surgery on intellectual function in infants and children. Cardiovasc Surg. 1996;4(3):303-7.

43. Hansen JH, Rotermann I, Logoteta J, Jung O, Dütschke P, Scheewe J, et al. Neurodevelopmental outcome in hypoplastic left heart syndrome: Impact of perioperative cerebral tissue oxygenation of the Norwood procedure. J Thorac Cardiovasc Surg. 2016;151(5):1358-66.

44. Heinrichs AK, Holschen A, Krings T, Messmer BJ, Schnitker R, Minkenberg R, et al. Neurologic and psycho-intellectual outcome related to structural brain imaging in adolescents and young adults after neonatal arterial switch operation for transposition of the great arteries. J Thorac Cardiovasc Surg. 2014;148(5):2190-9.

45. Heye KN, Rousson V, Knirsch W, Beck I, Liamlahi R, Bernet V, et al. Growth and Intellectual Abilities of Six-Year-Old Children with Congenital Heart Disease. J Pediatr. 2019;204:24-30.e10.

46. Hiraiwa A, Ibuki K, Tanaka T, Hirono K, Miya K, Yoshimura N, et al. Toddler Neurodevelopmental Outcomes Are Associated With School-Age IQ in Children With Single Ventricle Physiology. Semin Thorac Cardiovasc Surg. 2020;32(2):302-10.

47. Hiraiwa A, Kawasaki Y, Ibuki K, Hirono K, Matsui M, Yoshimura N, et al. Brain Development of Children With Single Ventricle Physiology or Transposition of the Great Arteries: A Longitudinal Observation Study. Semin Thorac Cardiovasc Surg. 2020;32(4):936-44.

48. Hofkosh D, Thompson AE, Nozza RJ, Kemp SS, Bowen A, Feldman HM. Ten years of extracorporeal membrane oxygenation: neurodevelopmental outcome. Pediatrics. 1991;87(4):549-55.

49. Hövels-Gürich HH, Seghaye MC, Schnitker R, Wiesner M, Huber W, Minkenberg R, et al. Long-term neurodevelopmental outcomes in school-aged children after neonatal arterial switch operation. J Thorac Cardiovasc Surg. 2002;124(3):448-58.

50. Ikle L, Hale K, Fashaw L, Boucek M, Rosenberg AA. Developmental outcome of patients with hypoplastic left heart syndrome treated with heart transplantation. J Pediatr. 2003;142(1):20-5.

51. Iwamoto I, Baba H, Koga Y, Uchida N, Matsuo K, Ishii K, et al. The relation between EEG and mental development following cardiac surgery performed under simple deep hypothermia in children. Jpn J Surg. 1990;20(2):158-62.

52. Jacobs A, Dulfer K, Eveleens RD, Hordijk J, Van Cleemput H, Verlinden I, et al. Long-term developmental effect of withholding parenteral nutrition in paediatric intensive care units: a 4-year follow-up of the PEPaNIC randomised controlled trial. Lancet Child Adolesc Health. 2020;4(7):503-14.

53. Jin Y, Liu J, Wang W, Wang Y, Yin Y, Xin X, et al. Neuropsychological development in school-aged children after surgery or transcatheter closure for ventricular septal defect. Neurol Sci. 2018;39(12):2053-60.

54. Jones B, Muscara F, Lloyd O, McKinlay L, Justo R. Neurodevelopmental outcome following open heart surgery in infancy: 6-year follow-up. Cardiol Young. 2015;25(5):903-10.

55. Karl TR, Hall S, Ford G, Kelly EA, Brizard CP, Mee RB, et al. Arterial switch with full-flow cardiopulmonary bypass and limited circulatory arrest: neurodevelopmental outcome. J Thorac Cardiovasc Surg. 2004;127(1):213-22.

56. Kaur J, Singhi P, Singhi S, Malhi P, Saini AG. Neurodevelopmental and Behavioral Outcomes in Children With Sepsis-Associated Encephalopathy Admitted to Pediatric Intensive Care Unit: A Prospective Case Control Study. J Child Neurol. 2016;31(6):683-90.

57. Kern JH, Hinton VJ, Nereo NE, Hayes CJ, Gersony WM. Early developmental outcome after the Norwood procedure for hypoplastic left heart syndrome. Pediatrics. 1998;102(5):1148-52.

58. King TZ, Smith KM, Burns TG, Sun B, Shin J, Jones RA, et al. fMRI investigation of working memory in adolescents with surgically treated congenital heart disease. Appl Neuropsychol Child. 2017;6(1):7-21.

59. Kirshbom PM, Flynn TB, Clancy RR, Ittenbach RF, Hartman DM, Paridon SM, et al. Late neurodevelopmental outcome after repair of total anomalous pulmonary venous connection. J Thorac Cardiovasc Surg. 2005;129(5):1091-7.

60. Krueger JJ, Brotschi B, Balmer C, Bernet V, Latal B. Postoperative Hyperglycemia and 4-Year Neurodevelopmental Outcome in Children Operated for Congenital Heart Disease. J Pediatr. 2015;167(6):1253-8.e1.

61. Krull K, Fuchs C, Yurk H, Boone P, Alonso E. Neurocognitive outcome in pediatric liver transplant recipients. Pediatr Transplant. 2003;7(2):111-8.

62. Langenbacher DN, T.; Poulsen, M.K. Neurodevelopmental Outcome of ECMO Survivors at Five Years of Age: The Potential for Academic and Motor Difficulties. 2001. p. 156-60.

63. Latal B, Wohlrab G, Brotschi B, Beck I, Knirsch W, Bernet V. Postoperative Amplitude-Integrated Electroencephalography Predicts Four-Year Neurodevelopmental Outcome in Children with Complex Congenital Heart Disease. J Pediatr. 2016;178:55-60.e1.

64. Latal B, Patel P, Liamlahi R, Knirsch W, O'Gorman Tuura R, von Rhein M. Hippocampal volume reduction is associated with intellectual functions in adolescents with congenital heart disease. Pediatr Res. 2016;80(4):531-7.

65. Leeuwen L, Schiller RM, Rietman AB, van Rosmalen J, Wildschut ED, Houmes RJM, et al. Risk Factors of Impaired Neuropsychologic Outcome in School-Aged Survivors of Neonatal Critical Illness. Crit Care Med. 2018;46(3):401-10.

66. Ma S, Li Y, Liu Y, Xu C, Li H, Yao Q, et al. Changes in Cortical Thickness Are Associated With Cognitive Ability in Postoperative School-Aged Children With Tetralogy of Fallot. Front Neurol. 2020;11:691.

67. Madderom MJ, Toussaint L, van der Cammen-van Zijp MH, Gischler SJ, Wijnen RM, Tibboel D, et al. Congenital diaphragmatic hernia with(out) ECMO: impaired development at 8 years. Arch Dis Child Fetal Neonatal Ed. 2013;98(4):F316-22.

68. Madderom MJ, Schiller RM, Gischler SJ, van Heijst AF, Tibboel D, Aarsen FK, et al. Growing Up After Critical Illness: Verbal, Visual-Spatial, and Working Memory Problems in Neonatal Extracorporeal Membrane Oxygenation Survivors. Crit Care Med. 2016;44(6):1182-90.

69. Mahle WT, Visconti KJ, Freier MC, Kanne SM, Hamilton WG, Sharkey AM, et al. Relationship of surgical approach to neurodevelopmental outcomes in hypoplastic left heart syndrome. Pediatrics. 2006;117(1):e90-7.

70. Majnemer A, Limperopoulos C, Shevell M, Rohlicek C, Rosenblatt B, Tchervenkov C. Developmental and functional outcomes at school entry in children with congenital heart defects. J Pediatr. 2008;153(1):55-60.

71. Melchers P, Maluck A, Suhr L, Scholten S, Lehmkuhl G. An Early Onset Rehabilitation Program for Children and Adolescents after Traumatic Brain Injury (TBI): Methods and First Results. Restor Neurol Neurosci. 1999;14(2-3):153-60.

72. Mesotten D, Gielen M, Sterken C, Claessens K, Hermans G, Vlasselaers D, et al. Neurocognitive development of children 4 years after critical illness and treatment with tight glucose control: a randomized controlled trial. Jama. 2012;308(16):1641-50.

73. Miatton M, De Wolf D, François K, Thiery E, Vingerhoets G. Do parental ratings on cognition reflect neuropsychological outcome in congenital heart disease? Acta Paediatr. 2008;97(1):41-5.

74. Mittnacht J, Choukair D, Kneppo C, Brunner R, Parzer P, Gorenflo M, et al. Long-Term Neurodevelopmental Outcome of Children Treated with Tri-Iodothyronine after Cardiac Surgery: Follow-Up of a Double-Blind, Randomized, Placebo-Controlled Study. Horm Res Paediatr. 2015;84(2):130-6.

75. Morris RD, Krawiecki NS, Wright JA, Walter LW. Neuropsychological, academic, and adaptive functioning in children who survive in-hospital cardiac arrest and resuscitation. J Learn Disabil. 1993;26(1):46-51.

76. Muñoz-López M, Hoskote A, Chadwick MJ, Dzieciol AM, Gadian DG, Chong K, et al. Hippocampal damage and memory impairment in congenital cyanotic heart disease. Hippocampus. 2017;27(4):417-24.

77. Murphy LK, Compas BE, Reeslund KL, Gindville MC, Mah ML, Markham LW, et al. Cognitive and attentional functioning in adolescents and young adults with Tetralogy of Fallot and d-transposition of the great arteries. Child Neuropsychol. 2017;23(1):99-110.

78. Naef N, Liamlahi R, Beck I, Bernet V, Dave H, Knirsch W, et al. Neurodevelopmental Profiles of Children with Congenital Heart Disease at School Age. J Pediatr. 2017;188:75-81.

79. Naguib AN, Winch PD, Tobias JD, Yeates KO, Miao Y, Galantowicz M, et al. Neurodevelopmental outcome after cardiac surgery utilizing cardiopulmonary bypass in children. Saudi J Anaesth. 2015;9(1):12-8.

80. Neufeld RE, Clark BG, Robertson CM, Moddemann DM, Dinu IA, Joffe AR, et al. Five-year neurocognitive and health outcomes after the neonatal arterial switch operation. J Thorac Cardiovasc Surg. 2008;136(6):1413-21, 21.e1-21.e2.

81. Nijhuis-van der Sanden MW, van der Cammen-van Zijp MH, Janssen AJ, Reuser JJ, Mazer P, van Heijst AF, et al. Motor performance in five-year-old extracorporeal membrane oxygenation survivors: a population-based study. Crit Care. 2009;13(2):R47.

82. Oates RK, Simpson JM, Turnbull JA, Cartmill TB. The relationship between intelligence and duration of circulatory arrest with deep hypothermia. J Thorac Cardiovasc Surg. 1995;110(3):786-92.

83. Oberhuber RD, Huemer S, Mair R, Sames-Dolzer E, Kreuzer M, Tulzer G. Cognitive Development of School-Age Hypoplastic Left Heart Syndrome Survivors: A Single Center Study. Pediatr Cardiol. 2017;38(6):1089-96.

84. Omeje IC, Hupka V, Kaldararova M, Ginzeriova M, Nosal M, Siman J, et al. Functional outcome of surgery for coarctation of the aorta. Bratisl Lek Listy. 2003;104(4-5):143-8.

85. Poncelet AJ, van Steenberghe M, Moniotte S, Detaille T, Beauloye C, Bertrand L, et al. Cardiac and neurological assessment of normothermia/warm blood cardioplegia vs hypothermia/cold crystalloid cardioplegia in pediatric cardiac surgery: insight from a prospective randomized trial. Eur J Cardiothorac Surg. 2011;40(6):1384-90.

86. Quartermain MD, Ittenbach RF, Flynn TB, Gaynor JW, Zhang X, Licht DJ, et al. Neuropsychological status in children after repair of acyanotic congenital heart disease. Pediatrics. 2010;126(2):e351-9.

87. Rotermann I, Logoteta J, Falta J, Wegner P, Jung O, Dütschke P, et al. Neuro-developmental outcome in single-ventricle patients: is the Norwood procedure a risk factor? Eur J Cardiothorac Surg. 2017;52(3):558-64.

88. Ryerson LM, Guerra GG, Joffe AR, Robertson CM, Alton GY, Dinu IA, et al. Survival and neurocognitive outcomes after cardiac extracorporeal life support in children less than 5 years of age: a ten-year cohort. Circ Heart Fail. 2015;8(2):312-21.

89. Sarajuuri A, Jokinen E, Puosi R, Eronen M, Mildh L, Mattila I, et al. Neurodevelopmental and neuroradiologic outcomes in patients with univentricular heart aged 5 to 7 years: related risk factor analysis. J Thorac Cardiovasc Surg. 2007;133(6):1524-32.

90. Sarajuuri A, Jokinen E, Mildh L, Tujulin AM, Mattila I, Valanne L, et al. Neurodevelopmental burden at age 5 years in patients with univentricular heart. Pediatrics. 2012;130(6):e1636-46.

91. Sarrechia I, Miatton M, François K, Gewillig M, Meyns B, Vingerhoets G, et al. Neurodevelopmental outcome after surgery for acyanotic congenital heart disease. Res Dev Disabil. 2015;45-46:58-68.

92. Sarrechia I, De Wolf D, Miatton M, François K, Gewillig M, Meyns B, et al. Neurodevelopment and behavior after transcatheter versus surgical closure of secundum type atrial septal defect. J Pediatr. 2015;166(1):31-8.

93. Schaefer C, von Rhein M, Knirsch W, Huber R, Natalucci G, Caflisch J, et al. Neurodevelopmental outcome, psychological adjustment, and quality of life in adolescents with congenital heart disease. Dev Med Child Neurol. 2013;55(12):1143-9.

94. Schiller RM, Madderom MJ, Reuser JJ, Steiner K, Gischler SJ, Tibboel D, et al. Neuropsychological Follow-up After Neonatal ECMO. Pediatrics. 2016;138(5).

95. Shida H, Morimoto M, Inokawa K, Ikeda Y, Tsugane J, Yuzuriha H. Somatic and psychomotor development of children after hypothermic open-heart surgery. Jpn J Surg. 1981;11(3):154-61.

96. Simons JS, Glidden R, Sheslow D, Pizarro C. Intermediate neurodevelopmental outcome after repair of ventricular septal defect. Ann Thorac Surg. 2010;90(5):1586-91.

97. Singer LT, Kercsmar C, Legris G, Orlowski JP, Hill BP, Doershuk C. Developmental sequelae of long-term infant tracheostomy. Dev Med Child Neurol. 1989;31(2):224-30.

98. Slomine BS, Silverstein FS, Christensen JR, Page K, Holubkov R, Dean JM, et al. Neuropsychological Outcomes of Children 1 Year After Pediatric Cardiac Arrest: Secondary Analysis of 2 Randomized Clinical Trials. JAMA Neurol. 2018;75(12):1502-10.

99. Sorensen LG, Neighbors K, Martz K, Zelko F, Bucuvalas JC, Alonso EM. Longitudinal study of cognitive and academic outcomes after pediatric liver transplantation. J Pediatr. 2014;165(1):65-72.e2.

100. Stein ML, Bruno JL, Konopacki KL, Kesler S, Reinhartz O, Rosenthal D. Cognitive outcomes in pediatric heart transplant recipients bridged to transplantation with ventricular assist devices. J Heart Lung Transplant. 2013;32(2):212-20.

101. Sugimoto A, Ota N, Ibuki K, Miyakoshi C, Murata M, Tosaka Y, et al. Risk factors for adverse neurocognitive outcomes in school-aged patients after the Fontan operation. Eur J Cardiothorac Surg. 2013;44(3):454-61; discussion 61.

102. Urschel S, Bond GY, Dinu IA, Moradi F, Conway J, Garcia-Guerra G, et al. Neurocognitive outcomes after heart transplantation in early childhood. J Heart Lung Transplant. 2018;37(6):740-8.

103. Uzark K, Lincoln A, Lamberti JJ, Mainwaring RD, Spicer RL, Moore JW. Neurodevelopmental outcomes in children with Fontan repair of functional single ventricle. Pediatrics. 1998;101(4 Pt 1):630-3.

104. Uzark K, Spicer R, Beebe DW. Neurodevelopmental outcomes in pediatric heart transplant recipients. J Heart Lung Transplant. 2009;28(12):1306-11.

105. van der Rijken R, Hulstijn-Dirkmaat G, Kraaimaat F, Nabuurs-Kohrman L, Nijveld A, Maassen B, et al. Open-heart surgery at school age does not affect neurocognitive functioning. Eur Heart J. 2008;29(21):2681-8.

106. Venchiarutti M, Vergine M, Zilli T, Sommariva G, Gortan AJ, Crescentini C, et al. Neuropsychological Impairment in Children With Class 1 Congenital Heart Disease. Percept Mot Skills. 2019;126(5):797-814.

107. Vergine M, Vedovelli L, Simonato M, Tonazzo V, Correani A, Cainelli E, et al. Perioperative Glial Fibrillary Acidic Protein Is Associated with Long-Term Neurodevelopment Outcome of Infants with Congenital Heart Disease. Children (Basel). 2021;8(8).

108. Vermunt LC, Buysse CM, Aarsen FK, Catsman-Berrevoets CE, Duivenvoorden HJ, Joosten KF, et al. Long-term cognitive functioning in children and adolescents who survived septic shock caused by Neisseria meningitidis. Br J Clin Psychol. 2009;48(Pt 2):195-208.

109. Vermunt LC, Buysse CM, Joosten KF, Duivenvoorden HJ, Hazelzet JA, Verhulst FC, et al. Survivors of septic shock caused by Neisseria meningitidis in childhood: psychosocial outcomes in young adulthood. Pediatr Crit Care Med. 2011;12(6):e302-9.

110. Volpe DSJ, Oliveira N, Santos AC, Linhares MBM, Carlotti A. Neuropsychological outcome of children with traumatic brain injury and its association with late magnetic resonance imaging findings: A cohort study. Brain Inj. 2017;31(12):1689-94.

111. von Rhein M, Dimitropoulos A, Valsangiacomo Buechel ER, Landolt MA, Latal B. Risk factors for neurodevelopmental impairments in school-age children after cardiac surgery with full-flow cardiopulmonary bypass. J Thorac Cardiovasc Surg. 2012;144(3):577-83.

112. Wells FC, Coghill S, Caplan HL, Lincoln C. Duration of circulatory arrest does influence the psychological development of children after cardiac operation in early life. J Thorac Cardiovasc Surg. 1983;86(6):823-31.

113. Wernovsky G, Stiles KM, Gauvreau K, Gentles TL, duPlessis AJ, Bellinger DC, et al. Cognitive development after the Fontan operation. Circulation. 2000;102(8):883-9.

114. Whitman V, Drotar D, Lambert S, VanHeeckeren DW, Borkat G, Ankeney J, et al. Effects of cardiac surgery with extracorporeal circulation on intellectual function in children. Circulation. 1973;48(1):160-3.

115. Wolfe KR, Kelly SL, Steinberg E, Pliego J, Everitt MD. Predictors of neuropsychological functioning and medication adherence in pediatric heart transplant recipients referred for neuropsychological evaluation. Pediatr Transplant. 2020;24(1):e13615.

116. Wolfe KR, Liptzin DR, Brigham D, Kelly SL, Rafferty C, Albertz M, et al. Relationships between Physiologic and Neuropsychologic Functioning after Fontan. J Pediatr. 2020;227:239-46.

117. Wotherspoon JM, Eagleson KJ, Gilmore L, Auld B, Hirst A, Johnson S, et al. Neurodevelopmental and health-related quality-of-life outcomes in adolescence after surgery for congenital heart disease in infancy. Dev Med Child Neurol. 2020;62(2):214-20.

118. Wray J, Pot-Mees C, Zeitlin H, Radley-Smith R, Yacoub M. Cognitive function and behavioural status in paediatric heart and heart-lung transplant recipients: the Harefield experience. Bmj. 1994;309(6958):837-41.

119. Wray J, Long T, Radley-Smith R, Yacoub M. Returning to school after heart or heart-lung transplantation: how well do children adjust? Transplantation. 2001;72(1):100-6.

120. Wray J, Sensky T. Congenital heart disease and cardiac surgery in childhood: effects on cognitive function and academic ability. Heart. 2001;85(6):687-91.

121. Wray J, Radley-Smith R. Beyond the first year after pediatric heart or heart-lung transplantation: Changes in cognitive function and behaviour. Pediatr Transplant. 2005;9(2):170-7.

122. Wray J, Radley-Smith R. Longitudinal assessment of psychological functioning in children after heart or heart-lung transplantation. J Heart Lung Transplant. 2006;25(3):345-52.

123. Wright M, Nolan T. Impact of cyanotic heart disease on school performance. Arch Dis Child. 1994;71(1):64-70.
